# Assessment of Variabilities in Lung-Contouring Methods on CBCT Preclinical Radiomics Outputs

**DOI:** 10.3390/cancers15102677

**Published:** 2023-05-09

**Authors:** Kathryn H. Brown, Jacob Illyuk, Mihaela Ghita, Gerard M. Walls, Conor K. McGarry, Karl T. Butterworth

**Affiliations:** 1 Patrick G. Johnston Centre for Cancer Research, Queen’s University Belfast, Belfast BT9 7AE, UKm.ghita@qub.ac.uk (M.G.); g.walls@qub.ac.uk (G.M.W.); conor.mcgarry@belfasttrust.hscni.net (C.K.M.); k.butterworth@qub.ac.uk (K.T.B.); 2 Northern Ireland Cancer Centre, Belfast Health & Social Care Trust, Belfast BT9 7JL, UK

**Keywords:** radiomics, preclinical analysis, lung, segmentation, inter-observer, ITK-SNAP, 3D Slicer

## Abstract

**Simple Summary:**

This study is the first to evaluate the impact of contouring differences on radiomics analysis in preclinical CBCT scans. We found that the variation in quantitative image readouts was greater between segmentation tools than between observers.

**Abstract:**

Radiomics image analysis has the potential to uncover disease characteristics for the development of predictive signatures and personalised radiotherapy treatment. Inter-observer and inter-software delineation variabilities are known to have downstream effects on radiomics features, reducing the reliability of the analysis. The purpose of this study was to investigate the impact of these variabilities on radiomics outputs from preclinical cone-beam computed tomography (CBCT) scans. Inter-observer variabilities were assessed using manual and semi-automated contours of mouse lungs (*n* = 16). Inter-software variabilities were determined between two tools (3D Slicer and ITK-SNAP). The contours were compared using Dice similarity coefficient (DSC) scores and the 95th percentile of the Hausdorff distance (HD_95p_) metrics. The good reliability of the radiomics outputs was defined using intraclass correlation coefficients (ICC) and their 95% confidence intervals. The median DSC scores were high (0.82–0.94), and the HD_95p_ metrics were within the submillimetre range for all comparisons. the shape and NGTDM features were impacted the most. Manual contours had the most reliable features (73%), followed by semi-automated (66%) and inter-software (51%) variabilities. From a total of 842 features, 314 robust features overlapped across all contouring methodologies. In addition, our results have a 70% overlap with features identified from clinical inter-observer studies.

## 1. Introduction

Radiomics is a novel image analysis approach which has the potential to revolutionise personalised diagnosis and treatment in oncology. Predictive models have been developed, including imaging biomarkers for protein expression, disease staging and survival, yet additional work is required to refine these models before they are deemed clinically acceptable [1,2,3,4].

Mouse models are ideal for developing new predictive radiomics signatures as they enable large-scale evaluations in a shorter timeline than clinical analyses [5,6]. Mouse models already exist, are widely used within oncology and are fundamental to our current understanding of tumour biology and cancer therapies [6]. Additionally, small-animal irradiators with onboard imaging for targeted exposures have revolutionised preclinical radiotherapy research [7]. These platforms provide the means to conduct small-animal imaging for the non-invasive visualisation of tissues that provides the potential to expand radiomics signatures that can be directly translatable for tumour diagnosis and the quantification of follow-up toxicities. Several recent studies have demonstrated the utility of such platforms for computed tomography (CT)- and magnetic resonance (MR)-based preclinical radiomics [8,9,10].

For a quantitative image analysis, a volume of tissue or tumour must be prospectively identified for analysis. There are two main segmentation methods: manual and automated. Manual contouring is a labour-intensive process and is affected by differences in delineation performance between users. In comparison, automated methods improve standardisation and workflow efficiency by automatically generating a draft region of interest [11,12]. Automated contours are typically good for larger tissues but are more prone to errors in smaller structures. Semi-automated methods are more desirable as they combine automation with user expertise, allowing users to alter automated methods and manually amend outputs if required [13].

Achieving accurate region-of-interest (ROI) contours for a tumour or normal tissue can be challenging due to limitations in scan resolution, motion management, and contrast enhancement. Where the tissue of interest is intimately related to another tissue of a similar CT density without a clear soft tissue plane, deciding a “ground truth” gold-standard representation can only be achieved through the consensus (and compromise) of experts. Furthermore, additional variation is introduced by the operator’s experience level, the soft tissue definition of the anatomical region in question and platform tool variations. Clinical studies have shown that these inter-observer variabilities have a direct impact on the reproducibility of radiomics features [14,15,16,17,18].

For the development of preclinical radiomics models, it is necessary to assess the reliability and robustness of radiomics features to differences in delineation. The predictive accuracy of these models is subject to user error in the segmentation of an ROI. The segmentation of tissues and tumours in preclinical models is further complicated by the small volumes involved and the limited resolution of preclinical cone-beam CT (CBCT) scans. Additional inter-observer variation and the absence of recommendations for the choice of contouring software used means that there is a high risk of errors in preclinical radiomics outputs. To progress the field, it would therefore be prudent for radiomics features robust to delineation variabilities to be determined.

The purpose of this study was to quantify and compare differences between manual and semi-automated contouring methods for lung segmentation on preclinical CBCT scans. We also aimed to assess the reliability and robustness of radiomics outputs to inter-observer variabilities across different software tools to elucidate stable radiomics features for future analyses.

## 2. Materials and Methods

### 2.1. CBCT Datasets

Preclinical CBCT scans were acquired on the Small Animal Radiation Research Platform (SARRP) (Xstrahl Life Sciences, Camberley, UK). These scans were part of a previous radiobiology study using female 8–12-week-old SCID mice. Pre-treatment CBCT scans (*n* = 16) were acquired at an imaging energy of 60 kV (using 0.5 mm Al filtration). Image reconstruction was performed using 360° images and filtered back-projection without postfiltering. Log(white/x) was applied to input images using FDK with a Hamming filtering window. CBCT scans had a slice thickness of 0.26 mm and were saved as DICOM files. All previous experimental procedures were carried out in accordance with the Home Office Guidance on the Operation of the Animals (Scientific Procedures) Act 1986 (PPL2813).

### 2.2. Contouring Methods

**Figure 1:**
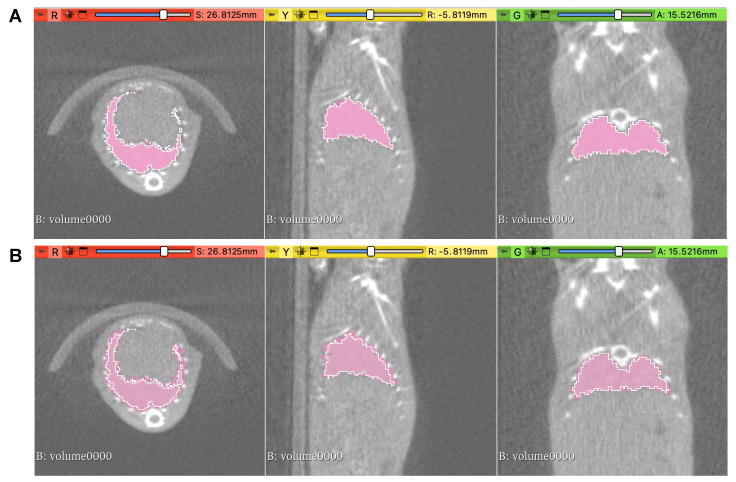
Examples of contours of mouse lungs from preclinical CBCT scans. (**A**) Contours created using semi-automated methods for two independent observers (pink fill and white outline) with a DSC score of 0.97 and HD_95p_ of 0.61. (**B**) Contours created by the same observer using 3D Slicer and ITK-SNAP (pink fill and white outline) with a DSC score of 0.84 and HD_95p_ of 0.79.

Examples of lung contours are shown in Figure 1. The contouring of the left and right lungs as one structure was performed by two independent, experienced observers (K.H.B. and J.I.). All contours were saved in 3D as NIfTI files (.nii). Manual contours of the lungs were created by both observers on 3D Slicer software (version 5.0.3, https://www.slicer.org (accessed on 26 July 2022)) using in-built paintbrush tools. Manual contours were created slice-by-slice in the coronal plane, with corrections made using other planes. Semi-automated contours were created using the “semi-automatic active contour segmentation” tool in ITK-SNAP software (version 3.8.0, http://www.itksnap.org (accessed on 26 July 2022)). Thresholding was used to differentiate the lungs from other surrounding structures. To initiate contouring, “bubbles” were placed in multiple areas of the lungs in three planes to aid in the creation of contours. After manual inspection, this process was altered and repeated until the observers were satisfied that it was an accurate representation of the lungs.

For inter-software analysis, the lungs were contoured manually slice-by-slice by one observer (J.I.) using 3D Slicer and ITK-SNAP tools. In-built brush tools were used, with contours created using the coronal plane and corrections made using the sagittal and axial planes.

### 2.3. Radiomics Analysis

Radiomics feature extraction was completed using PyRadiomics software (version 2.7.7) [19]. Features were extracted with a resampled pixel spacing of 0.26 mm and a fixed bin width of 25. Wavelet filtering was applied with both the unfiltered (original) and filtered (wavelet) features extracted for analysis. A total of 842 features were extracted and classified by the following feature classes: morphology (shape) (*n* = 14), intensity (first order) (*n* = 18) and texture (grey level cooccurrence matrix (GLCM) (*n* = 23), grey level run length matrix (GLRLM) (*n* = 16), grey level size zone matrix (GLSZM) (*n* = 16), grey level dependence matrix (GLDM) (*n* = 14), and neighbouring grey tone difference matrix (NGTDM) (*n* = 5)). Further details on these features can be found in the Image Biomarker Standardisation Initiative (IBSI) [19,20].

### 2.4. Statistical Analysis

The statistical analysis was completed using GraphPad Prism (version 7.01), RStudio software (version 4.1.2) and 3D Slicer (version 5.0.3, https://www.slicer.org (accessed on 26 July 2022)). SlicerRT within the 3D Slicer software was used to calculate the Dice similarity coefficient (DSC) and Hausdorff distance (HD) metrics. The DSC scores were calculated to compare the overlap of the lung contours in which 1 indicates perfect overlap and 0 indicates no overlap [21]. The HD metrics were calculated as a percentile of the distances between the surface of one segmentation to another [22]. The 95th percentile of the Hausdorff distance (HD_95p_) was calculated to avoid the impact of outliers. Median and standard deviation (SD) values (*n* = 16) were calculated in GraphPad Prism.

The intraclass correlation coefficient (ICC) was used to assess the level of reliability and the robustness of the features. ICCs were calculated using the *irr* library (*lpSolve* package, RStudio software (version 4.1.2)), using the 2-way random-effects model. An ICC of 0 indicates no reliability, and an ICC of 1 indicates perfect reliability. ICC outputs are classified by Koo et al. as poor (<0.5), moderate (0.5–0.7), good (0.7–0.9) and excellent (>0.9) [23]. Further analysis was conducted to determine the robustness of these results. Robustness was assessed using the lower 95% confidence intervals (CI) of the ICC (i.e., the worst-case scenario receives a lower reliability score), with a lower CI ICC of >0.7 used to identify good robustness [23,24].

## 3. Results

### 3.1. Geometry

**Figure 2:**
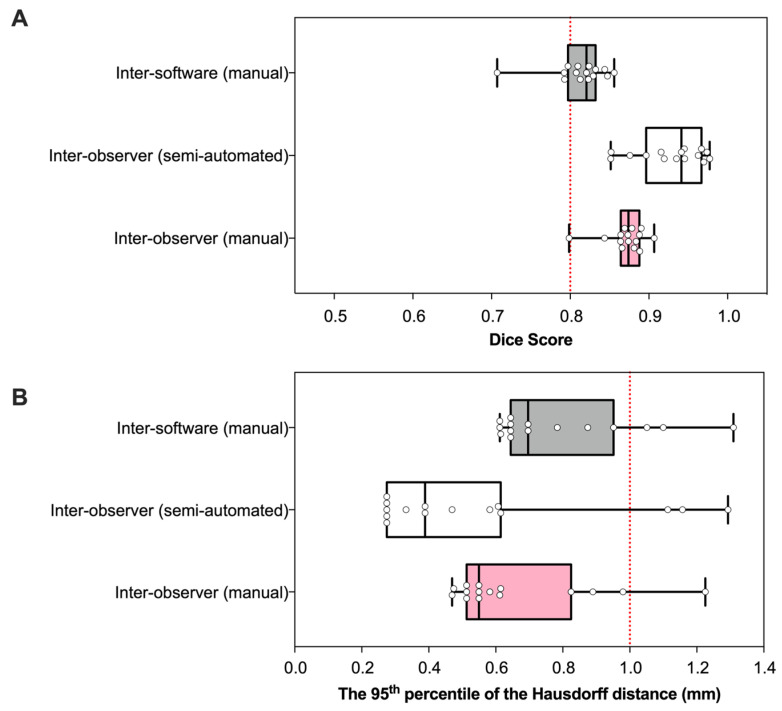
Similarity of manual and semi-automated contours. Boxplots show Dice similarity coefficients (DSC) (**A**) and the 95th percentile of the Hausdorff distances (HD_95p_) (**B**) for lungs contoured using manual and semi-automated methods.

The DSC and HD_95p_ values were used to compare the inter-observer and software differences for the contouring of mouse lungs on CBCT scans (Figure 2). All DSC scores were greater than 0.8, indicating good overlap. The median DSC score ± SD for semi-automated contours was higher (0.95 ± 0.04) in comparison to the manual contours (0.87 ± 0.02) and software differences (0.82 ± 0.04) (Figure 2A). The HD_95p_ metrics also suggest that the semi-automated contours had the greatest similarity between observers, as evidenced by the smallest median of 0.39 mm ± 0.35. Manual contours had a marginally higher median HD_95p_ value of 0.55 mm ± 0.22, yet most values were within the submillimetre range. Inter-software comparisons of manual contours had the highest HD_95p_ value of 0.70 mm ± 0.22 (Figure 2B).

Figure 1 shows an example of contours with DSC scores and HD_95p_ values of 0.97 and 0.61 (Panel A) in comparison to 0.84 and 0.7 (Panel B). Despite these variations, visually, the contours have good overlap, and differences may only be significant at the bordering points. Although a good starting point, the DSC and HD_95p_ metrics are not sufficient to compare delineation differences, and the similarity of the 3D shape, intensity and textural radiomics features is essential.

### 3.2. Reliability

**Figure 3:**
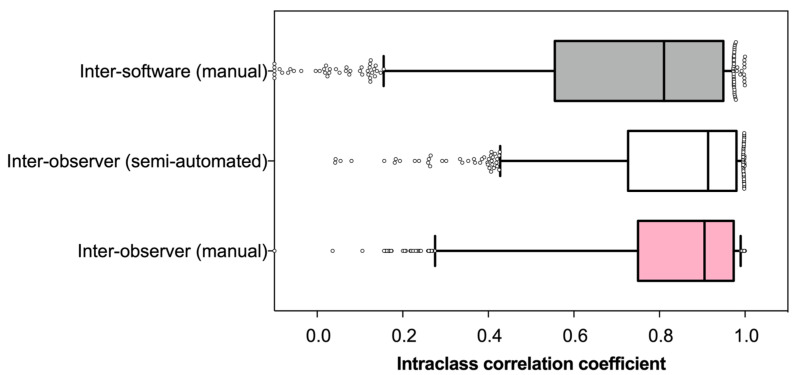
Distribution of the intraclass correlation coefficients of radiomics features for different contouring methods.

ICCs were calculated to determine the reliability of the features for different contouring methods and software. Inter-observer contouring methods (manual and semi-automated) had median ICC values ± SD of 0.91 ± 0.21 and 0.91 ± 0.19 respectively, indicating an “excellent” level of reliability between observers across both contouring methods. The median ICC for manual contours on different software was slightly lower at 0.81 ± 0.28 but still within the “good” reliability ranking (Figure 3). The manual inter-observer contours had the most reliable features of 611 features (73%). In comparison, the semi-automated inter-observer contours had 555 (66%), and the software comparison had 431 reliable features (51%).

**Figure 4:**
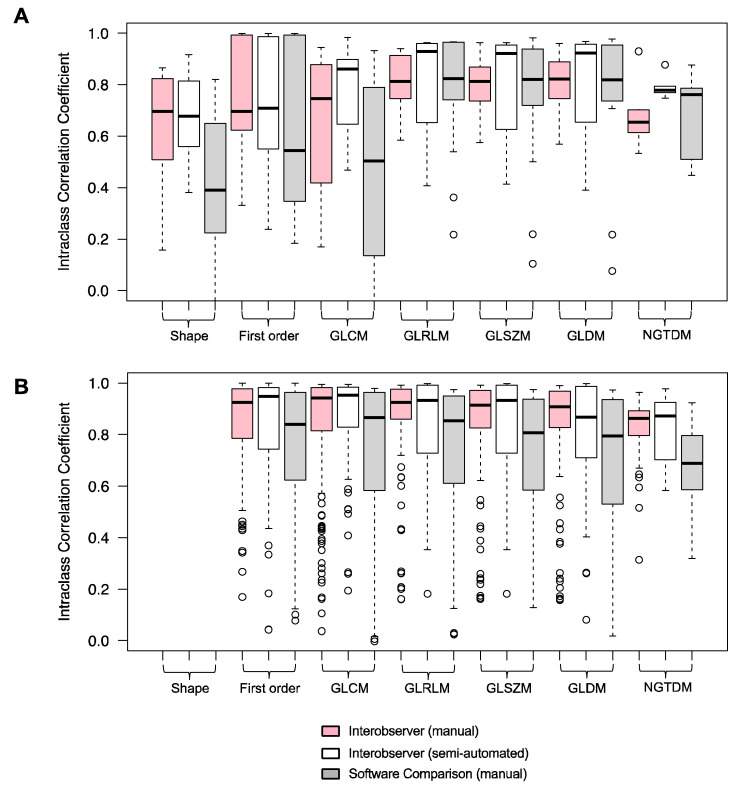
Boxplots of intraclass correlation coefficients by feature class and feature type (unfiltered (**A**) and filtered (wavelet) (**B**)).

The ICC values varied according to feature classes (shape, intensity and texture) (Figure 4). For unfiltered features, the median ICC values were the highest for texture features (GLRLM, GLSZM and GLDM) and were lower for the shape and intensity (first order) features (Figure 4A). The semi-automated methods consistently had the highest median ICC values for GLRLM, GLSZM and GLDM of 0.93, 0.92 and 0.92, respectively. Shape features were affected the most by inter-observer contouring differences, with the lowest median ICC values of 0.70 ± 0.30 (manual) and 0.68 ± 0.17 (semi-automated). Inter-software variabilities had the greatest impact on shape features with the lowest median ICC of 0.39 ± 0.29, indicating “poor” reliability.

Wavelet filtering increased the median ICC value for most feature classes (Figure 4B). The largest increase was seen for the first-order and texture features. All filtered features had a median ICC > 0.8 except for the NGTDM features for inter-software comparisons, which had a median ICC of 0.69 ± 0.15. Overall, the inter-software results had the largest spread of ICC values, indicating reduced reliability in comparison to the intra-software results.

**Figure 5:**
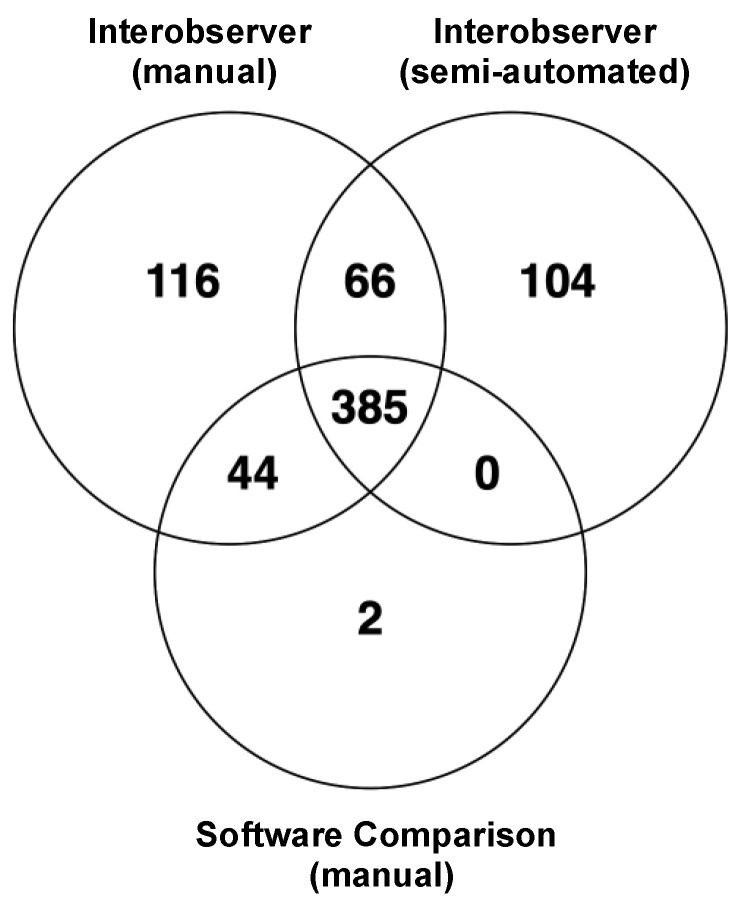
Overlapping features with an ICC > 0.8 across different contouring methods, comprised of a total 28 unfiltered and 357 filtered (wavelet) features.

Of the 842 features analysed, 385 features had an ICC > 0.8 for all comparisons (Figure 5). These included 1 shape, 79 intensity and 305 texture (110 GLCM, 69 GLRLM, 64 GLSZM, 52 GLDM and 10 NGTDM) features. The majority of overlapping unfiltered features were first-order, GLRLM and GLSZM features, whereas for filtered features, the majority were GLCM features.

### 3.3. Robustness

**Figure 6:**
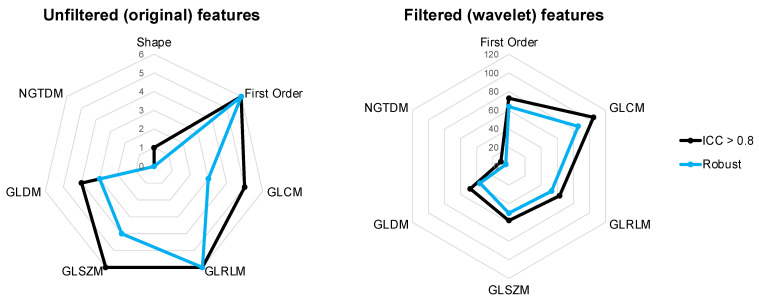
Spider plots to show the distribution of features with an ICC > 0.8 per feature class. A total of 314 features were robust, with a lower confidence interval of >0.7 (22 unfiltered and 292 filtered).

To account for potential errors and ensure the results were robust, the lower 95% CIs of the ICC were analysed [23]. All contouring comparisons had a high percentage (76–78%) of reliable features which were also robust. Of the 385 overlapping features, 314 features were also robust (Figure 6). These included 70 intensity and 244 texture (88 GLCM, 59 GLRLM, 54 GLSZM, 40 GLDM and 3 NGTDM) features (Supplementary Tables S1 and S2). NGTDM features were impacted the most by differences in contouring methods, with a lower median of 0.63 ± 0.10, and only 40% of the reliable features were also considered robust. The first-order, GLSZM, GLRLM and GLCM feature classes had 88%, 86%, 84% and 82% of robust features which were also reliable and therefore more stable for analysis.

## 4. Discussion

Radiomics analysis is paving the way for precision medicine through the improvement of diagnosis and prognosis within oncology. Encouragingly, there are more publicly available clinical datasets that can be used for analysis, but still there is a lack of “big” and standardised data that can be used to create meaningful radiomics signatures. Standardisation is limited by different image acquisition protocols, variabilities in patient history and restrictions by law and ethics [25], yet preclinical models pose an alternative source for developing this analysis in controlled scenarios.

Radiomics workflows are comprised of four main steps: image acquisition, tissue delineation, feature extraction and statistical analysis. The reproducibility of this workflow is dependent on each step being standardised. Some steps are easier to standardise (image acquisition and feature extraction), whilst others will be dependent on the objective of the study (statistical analysis) or user variability (tissue delineation) [26]. It is recommended to standardise all possible steps within the workflow, including imaging modality, imaging energy, image intensity discretisation, image processing, image normalisation methods, radiomics software used for the extraction of features and the filtering of features [20,27,28,29]. These, among many others are, essential factors to standardise and ensure that results are robust and reproducible. However, tissue delineation will always lead to variabilities in radiomics outputs. This study is the first to evaluate the influence of inter-observer (manual and semi-automated) and inter-software variabilities on preclinical radiomics results.

Accuracy in the delineation of an ROI is an essential step in generating meaningful results. Previous studies have shown that variations in contouring or inter-observer delineation can influence radiomics features [15,30,31]. Some tissues are more susceptible to changes in radiomics features, with the greater variability detected for lower-contrast tissues restricting the number of robust features available for analysis [16,17]. A reduction of error from the true volume of the ROI is also particularly important during radiomics analysis due to “the volume effect” phenomenon. Some studies suggest the predictive ability of a radiomics signature is often correlated to the volume of the ROI [32,33]. Inter-observer variations, which largely alter the volume, may therefore have a significant effect on radiomics analysis. We suspect this may be particularly applicable in preclinical radiomics models due to their small size and the lack of standardisation in segmentation methods.

The introduction of automation could not only reduce the time-consuming process of manual contouring but also reduce human variations, in theory leading to more reproducible contours [12,34]. Studies have shown that comparable results can be produced from observers with different levels of contouring expertise when using semi-automated contouring methods [31], yet other studies have also shown that the complexity and irregular pattern of a tissue will largely influence the similarity of contours [15].

Our results show that inter-observer variability was reduced by using semi-automated contouring methods in comparison to manual contouring, as evidenced through higher DSC and lower HD_95p_ values. Despite having fewer stable features, semi-automated contours had “excellent” reliability for most feature classes. This suggests that the semi-automated contouring of the lung may provide a quality assurance step in radiomics analysis. Other studies have shown similar findings, with improved feature reliability for the semi-automated contours of tumours [30,31].

Some studies have reported that semi-automated and automated contouring algorithms, within the same software or between different software packages, may not be suitable to be used interchangeably due to differences in the repeatability and reproducibility of contours [31,35,36]. Our results emphasised the importance of identifying differences in contouring software for radiomics analysis to improve comparability of results.

Inter-software variations reduced the number of robust radiomics features for analysis. This could be due to multiple factors, including the compatibility of CBCT-data-loading (DICOM) and segmentation-saving (NIfTI) formats, paintbrush size and daily intra-observer variations. As we used no algorithms or automation within the software comparison, we suspect inter-software variations are an accumulation of these and can be minimised in future by using one contouring software tool. We acknowledge that this may not be possible for multi-centre analysis, but in this study, we identified 431 radiomics features with good reliability across both Slicer and ITK-SNAP. Of these, 77% features are highly robust.

Across all analysis, we found the intensity (first-order) and texture (GLSZM, GLRLM and GLCM) features to be the most robust and reliable. The shape and NGTDM features were impacted the most by differences in contouring methods. We suspect that shape features were not stable as they are defined by the geometry of the ROI, which in this study, was the dependent variable. Similar to other clinical studies, we found that applying wavelet filtering increased the median ICC values for most radiomics feature classes [14]. This could be a solution for removing contouring variations and improving the repeatability of radiomics outputs.

Encouragingly, even these first data are relatable to clinical studies [14,15]. No studies could be found to document the impact of inter-observer variabilities on radiomics features in the lung; therefore, similarities were drawn from tumour examples. Pavic et al. evaluated the impact of inter-observer delineation in non-small cell lung cancer (NSCLC) tumours [15]. In comparison to our stable features, 277 features (72%) were also found to be stable with the data from Pavic et al. (Supplementary Table S3). More recently, Kothari et al. assessed the robustness of inter-observer variation in another cohort of NSCLC tumours [14]. Only 54 features (unfiltered and wavelet) were listed with an ICC < 0.6, and just 7 of these were identified as unfavourable features within our study, suggesting good differentiation between stable and unstable features across both clinical and preclinical radiomics (Supplementary Table S4). From these comparisons, we identified 271 radiomics features which are robust to different segmentation methods (Supplementary Table S5) [14,15]. These include 14 unfiltered and 257 filtered features, with the first-order and texture feature classes being the most robust. This complementary analysis also strengthens the argument for the use of preclinical radiomics analysis to expand clinical radiomics signatures. Future work is required for the expansion of these preclinical radiomics models and to further the development of informative radiomics signatures.

We acknowledge that our study has several limitations. Firstly, the use of data from a single scanner, and only two observers were used for inter-observer assessments. To build on this pilot study with novel findings, a multi-centre preclinical investigation with a range of contouring experience would further validate the reliable features identified. We also acknowledge that in recent years, there has been the development of fully automated contouring methods for preclinical models [12,37], yet these are not widely available or fully adopted across preclinical imaging centres. We suspect the implementation of these into preclinical radiomics workflows will reduce variabilities and increase the robustness of features. Finally, preclinical CBCT scans contain more noise than clinical scans, which may influence the radiomics features. A test–retest analysis may overcome this to further determine reliable radiomics features for analysis, but this lay out of the scope of this manuscript. We hope that this work will enhance preclinical radiomics models so that they may be integrated more heavily in oncology studies to expand to imaging biomarkers.

## 5. Conclusions

The standardisation of workflows is vital to ensure the reproducibility of radiomics analyses including variations in the creation of ROIs. Our results are the first to evaluate the impact of contouring differences on preclinical radiomics. Although we showed that manual contouring provides more reliable features for analysis, semi-automated methods reduced variations, resulting in improved robustness of the radiomics readouts. Across all delineation variabilities, there were 314 robust imaging features. Our findings are clinically translatable, with a considerable proportion of features having been previously shown to have utility in large patient datasets. Ultimately, our findings suggest that automated contouring tools for preclinical models will reduce inter-observer and inter-software variabilities. This could significantly improve standardisation in preclinical radiomics workflows, which will directly impact the repeatability and reproducibility of predictive radiomics outputs.

## Data Availability

Datasets and analyses are available from the corresponding author.

## Supplementary Materials

The following supporting information can be downloaded at: https://www.mdpi.com/article/10.3390/cancers15102677/s1, Table S1: The robustness of radiomics features found to be reliable in all three contouring comparisons; Table S2: List of robust and reliable radiomics features by feature class; Table S3: Comparison of preclinical and clinical interobserver delineation variations in radiomics analysis; Table S4: Comparison of radiomics features from preclinical and clinical inter-observer delineation variability studies; Table S5: Radiomics features stable across three independent studies which assessed different methods of contouring of lung tissue and tumours and in both clinical and preclinical models.
