# Supplementary material for: Assessment of Variabilities in Lung-Contouring Methods on CBCT Preclinical Radiomics Outputs
Source: Cancers (Basel). 2023 May 9;15(10):2677. doi: 10.3390/cancers15102677 (PMC10216427; doi:10.3390/cancers15102677)
Supplement: Supplementary file 1 [file cancers-15-02677-s001.zip › cancers-2333786-supplementary.pdf]

cancers

MDPI

Assessment of Variabilities in Lung Contouring Methods on CBCT Preclinical Radiomics Outputs

Kathryn H. Brown, Jacob Illyuk, Mihaela Ghita, Gerard M. Walls, Conor K. McGarry and Karl T. Butterworth

Table S1. The robustness of radiomics features found to be reliable in all three contouring comparisons. Radiomics features which were reliable (ICC > 0.8) for all contouring comparisons were further analysed using the lower CI and those >0.7 were determined as robust (marked with an x). Features highlighted in blue represent those which are robust across all.

			Lower CI of ICC >0.7		
	Radiomics feature	ICC >0.8	Manual	Auto	Inter-software
Original (unfiltered)	original_shape_LeastAxisLength	x			
	original_firstorder_Minimum	x	x	x	x
	original_firstorder_10Percentile	x	x	x	x
	original_firstorder_90Percentile	x	x	x	x
	original_firstorder_Mean	x	x	x	x
	original_firstorder_Median	x	x	x	x
	original_firstorder_RootMeanSquared	x	x	x	x
	original_glcm_DifferenceAverage	x	x		
	original_glcm_DifferenceEntropy	x	x		
	original_glcm_Idm	x	x	x	x
	original_glcm_Id	x	x	x	x
	original_glcm_InverseVariance	x	x	x	x
	original_glrlm_GrayLevelNonUniformity	x	x	x	x
	original_glrlm_LongRunEmphasis	x	x	x	x
	original_glrlm_RunLengthNonUniformityNormalized	x	x	x	x
	original_glrlm_RunPercentage	x	x	x	x
	original_glrlm_RunVariance	x	x	x	x
	original_glrlm_ShortRunEmphasis	x	x	x	x
	original_glszm_GrayLevelNonUniformity	x	x	x	x
	original_glszm_LargeAreaEmphasis	x	x	x	x
	original_glszm_SizeZoneNonUniformityNormalized	x		x	x
	original_glszm_SmallAreaEmphasis	x		x	x
	original_glszm_ZonePercentage	x	x	x	x
	original_glszm_ZoneVariance	x	x	x	x
	original_gldm_DependenceNonUniformityNormalized	x	x	x	x
	original_gldm_DependenceVariance	x	x	x	x
	original_gldm_GrayLevelNonUniformity	x	x	x	x
	original_gldm_SmallDependenceEmphasis	x		x	x
Wavelet (filtered)	wavelet.LLH_firstorder_Entropy	x	x	x	x
	wavelet.LLH_firstorder_10Percentile	x	x	x	x
	wavelet.LLH_firstorder_90Percentile	x	x	x	x
	wavelet.LLH_firstorder_InterquartileRange	x	x	x	x

wavelet.LLH_firstorder_MeanAbsoluteDeviation	x	x	x	x
wavelet.LLH_firstorder_RobustMeanAbsoluteDeviation	x	x	x	x
wavelet.LLH_firstorder_RootMeanSquared	x		x	
wavelet.LLH_firstorder_Variance	x	x	x	
wavelet.LLH_firstorder_Uniformity	x	x	x	x
wavelet.LLH_glcm_ClusterTendency	x	x	x	
wavelet.LLH_glcm_Contrast	x	x	x	x
wavelet.LLH_glcm_DifferenceAverage	x	x	x	x
wavelet.LLH_glcm_DifferenceEntropy	x	x	x	x
wavelet.LLH_glcm_DifferenceVariance	x	x	x	x
wavelet.LLH_glcm_JointEnergy	x	x	x	x
wavelet.LLH_glcm_JointEntropy	x	x	x	x
wavelet.LLH_glcm_Imc1	x			
wavelet.LLH_glcm_Imc2	x	x		
wavelet.LLH_glcm_Idm	x	x	x	x
wavelet.LLH_glcm_Id	x	x	x	x
wavelet.LLH_glcm_InverseVariance	x	x	x	x
wavelet.LLH_glcm_MaximumProbability	x	x	x	x
wavelet.LLH_glcm_SumEntropy	x	x	x	
wavelet.LLH_glcm_SumSquares	x	x	x	
wavelet.LLH_glrlm_GrayLevelNonUniformityNormalized	x	x	x	x
wavelet.LLH_glrlm_GrayLevelVariance	x	x	x	
wavelet.LLH_glrlm_LongRunEmphasis	x	x	x	x
wavelet.LLH_glrlm_RunEntropy	x	x	x	
wavelet.LLH_glrlm_RunLengthNonUniformityNormalized	x	x	x	x
wavelet.LLH_glrlm_RunPercentage	x	x	x	x
wavelet.LLH_glrlm_RunVariance	x	x	x	x
wavelet.LLH_glrlm_ShortRunEmphasis	x	x	x	x
wavelet.LLH_glszm_GrayLevelNonUniformityNormalized	x	x	x	x
wavelet.LLH_glszm_GrayLevelVariance	x	x	x	
wavelet.LLH_glszm_LargeAreaEmphasis	x	x	x	x
wavelet.LLH_glszm_SizeZoneNonUniformityNormalized	x	x	x	x
wavelet.LLH_glszm_SmallAreaEmphasis	x	x	x	x
wavelet.LLH_glszm_ZonePercentage	x	x	x	x
wavelet.LLH_glszm_ZoneVariance	x	x	x	x
wavelet.LLH_gldm_DependenceNonUniformityNormalized	x	x	x	x
wavelet.LLH_gldm_DependenceVariance	x	x	x	x
wavelet.LLH_gldm_GrayLevelVariance	x	x	x	
wavelet.LLH_gldm_LargeDependenceEmphasis	x	x	x	x
wavelet.LLH_gldm_SmallDependenceEmphasis	x	x	x	x

wavelet.LHL_firstorder_Entropy	x	x	x	x
wavelet.LHL_firstorder_10Percentile	x	x	x	
wavelet.LHL_firstorder_90Percentile	x	x	x	x
wavelet.LHL_firstorder_Maximum	x	x	x	x
wavelet.LHL_firstorder_Median	x	x	x	
wavelet.LHL_firstorder_InterquartileRange	x	x	x	x
wavelet.LHL_firstorder_MeanAbsoluteDeviation	x	x	x	x
wavelet.LHL_firstorder_RobustMeanAbsoluteDeviation	x	x	x	x
wavelet.LHL_firstorder_RootMeanSquared	x	x	x	x
wavelet.LHL_firstorder_Variance	x	x	x	
wavelet.LHL_firstorder_Uniformity	x	x	x	x
wavelet.LHL_glcm_ClusterTendency	x	x	x	
wavelet.LHL_glcm_Contrast	x	x	x	
wavelet.LHL_glcm_Correlation	x	x	x	
wavelet.LHL_glcm_DifferenceAverage	x	x	x	x
wavelet.LHL_glcm_DifferenceEntropy	x	x	x	x
wavelet.LHL_glcm_DifferenceVariance	x	x		
wavelet.LHL_glcm_JointEnergy	x	x	x	x
wavelet.LHL_glcm_JointEntropy	x	x	x	x
wavelet.LHL_glcm_Imc1	x	x	x	
wavelet.LHL_glcm_Idm	x	x	x	x
wavelet.LHL_glcm_Id	x	x	x	x
wavelet.LHL_glcm_InverseVariance	x	x	x	x
wavelet.LHL_glcm_MaximumProbability	x	x	x	x
wavelet.LHL_glcm_SumEntropy	x	x	x	x
wavelet.LHL_glcm_SumSquares	x	x	x	
wavelet.LHL_glrlm_GrayLevelNonUniformity	x	x		
wavelet.LHL_glrlm_GrayLevelNonUniformityNormalized	x	x	x	x
wavelet.LHL_glrlm_GrayLevelVariance	x	x	x	
wavelet.LHL_glrlm_LongRunEmphasis	x	x	x	x
wavelet.LHL_glrlm_RunEntropy	x	x	x	x
wavelet.LHL_glrlm_RunLengthNonUniformityNormalized	x	x	x	x
wavelet.LHL_glrlm_RunPercentage	x	x	x	x
wavelet.LHL_glrlm_RunVariance	x	x	x	x
wavelet.LHL_glrlm_ShortRunEmphasis	x	x	x	x
wavelet.LHL_glszm_GrayLevelNonUniformityNormalized	x	x	x	x
wavelet.LHL_glszm_GrayLevelVariance	x	x	x	
wavelet.LHL_glszm_LargeAreaEmphasis	x	x	x	x
wavelet.LHL_glszm_SizeZoneNonUniformityNormalized	x	x	x	x
wavelet.LHL_glszm_SmallAreaEmphasis	x	x	x	x
wavelet.LHL_glszm_ZonePercentage	x	x	x	x
wavelet.LHL_glszm_ZoneVariance	x	x	x	x

wavelet.LHL_gldm_DependenceEntropy	x	x		
wavelet.LHL_gldm_DependenceNonUniformityNormalized	x	x	x	x
wavelet.LHL_gldm_DependenceVariance	x	x	x	x
wavelet.LHL_gldm_GrayLevelNonUniformity	x	x		
wavelet.LHL_gldm_GrayLevelVariance	x	x	x	
wavelet.LHL_gldm_LargeDependenceEmphasis	x	x	x	x
wavelet.LHL_gldm_SmallDependenceEmphasis	x	x	x	x
wavelet.LHL_ngtdm_Contrast	x		x	
wavelet.LHH_firstorder_Entropy	x	x	x	x
wavelet.LHH_firstorder_10Percentile	x	x	x	x
wavelet.LHH_firstorder_90Percentile	x	x	x	x
wavelet.LHH_firstorder_InterquartileRange	x	x	x	x
wavelet.LHH_firstorder_MeanAbsoluteDeviation	x	x	x	x
wavelet.LHH_firstorder_RobustMeanAbsoluteDeviation	x	x	x	x
wavelet.LHH_firstorder_Variance	x	x	x	
wavelet.LHH_firstorder_Uniformity	x	x	x	x
wavelet.LHH_glcm_ClusterTendency	x	x	x	x
wavelet.LHH_glcm_Contrast	x	x	x	x
wavelet.LHH_glcm_DifferenceAverage	x	x	x	x
wavelet.LHH_glcm_DifferenceEntropy	x	x	x	x
wavelet.LHH_glcm_DifferenceVariance	x	x		
wavelet.LHH_glcm_JointEnergy	x	x	x	x
wavelet.LHH_glcm_JointEntropy	x	x	x	x
wavelet.LHH_glcm_Idm	x	x	x	x
wavelet.LHH_glcm_Id	x	x	x	x
wavelet.LHH_glcm_InverseVariance	x	x	x	x
wavelet.LHH_glcm_MaximumProbability	x	x	x	x
wavelet.LHH_glcm_SumEntropy	x	x	x	x
wavelet.LHH_glcm_SumSquares	x	x	x	x
wavelet.LHH_glrlm_GrayLevelNonUniformityNormalized	x	x	x	x
wavelet.LHH_glrlm_GrayLevelVariance	x	x		
wavelet.LHH_glrlm_LongRunEmphasis	x	x	x	x
wavelet.LHH_glrlm_RunEntropy	x	x	x	x
wavelet.LHH_glrlm_RunLengthNonUniformityNormalized	x	x	x	x
wavelet.LHH_glrlm_RunPercentage	x	x	x	x
wavelet.LHH_glrlm_RunVariance	x	x	x	x
wavelet.LHH_glrlm_ShortRunEmphasis	x	x	x	x
wavelet.LHH_glszm_GrayLevelNonUniformityNormalized	x	x	x	x
wavelet.LHH_glszm_GrayLevelVariance	x	x		
wavelet.LHH_glszm_LargeAreaEmphasis	x	x	x	x

wavelet.LHH_glszm_SizeZoneNonUniformityNormalized	x	x	x	x
wavelet.LHH_glszm_SmallAreaEmphasis	x	x	x	x
wavelet.LHH_glszm_ZonePercentage	x	x	x	x
wavelet.LHH_glszm_ZoneVariance	x	x	x	x
wavelet.LHH_gldm_DependenceEntropy	x	x		
wavelet.LHH_gldm_DependenceNonUniformityNormalized	x	x	x	x
wavelet.LHH_gldm_DependenceVariance	x	x	x	x
wavelet.LHH_gldm_GrayLevelVariance	x	x	x	
wavelet.LHH_gldm_LargeDependenceEmphasis	x	x	x	x
wavelet.LHH_gldm_SmallDependenceEmphasis	x	x	x	x
wavelet.HLL_firstorder_Entropy	x	x	x	x
wavelet.HLL_firstorder_10Percentile	x	x	x	x
wavelet.HLL_firstorder_90Percentile	x	x	x	x
wavelet.HLL_firstorder_InterquartileRange	x	x	x	x
wavelet.HLL_firstorder_MeanAbsoluteDeviation	x	x	x	x
wavelet.HLL_firstorder_RobustMeanAbsoluteDeviation	x	x	x	x
wavelet.HLL_firstorder_RootMeanSquared	x	x	x	x
wavelet.HLL_firstorder_Variance	x	x	x	x
wavelet.HLL_firstorder_Uniformity	x	x	x	x
wavelet.HLL_glcm_ClusterProminence	x	x	x	
wavelet.HLL_glcm_ClusterTendency	x	x	x	x
wavelet.HLL_glcm_Contrast	x	x	x	x
wavelet.HLL_glcm_Correlation	x			
wavelet.HLL_glcm_DifferenceAverage	x	x	x	x
wavelet.HLL_glcm_DifferenceEntropy	x	x	x	x
wavelet.HLL_glcm_DifferenceVariance	x	x	x	x
wavelet.HLL_glcm_JointEnergy	x	x	x	x
wavelet.HLL_glcm_JointEntropy	x	x	x	x
wavelet.HLL_glcm_Imc1	x	x		
wavelet.HLL_glcm_Imc2	x	x		
wavelet.HLL_glcm_Idm	x	x	x	x
wavelet.HLL_glcm_Id	x	x	x	x
wavelet.HLL_glcm_InverseVariance	x	x	x	x
wavelet.HLL_glcm_MaximumProbability	x	x		
wavelet.HLL_glcm_SumEntropy	x	x	x	x
wavelet.HLL_glcm_SumSquares	x	x	x	x
wavelet.HLL_glrlm_GrayLevelNonUniformityNormalized	x	x	x	x
wavelet.HLL_glrlm_GrayLevelVariance	x	x	x	x
wavelet.HLL_glrlm_LongRunEmphasis	x	x	x	x
wavelet.HLL_glrlm_RunEntropy	x	x	x	x
wavelet.HLL_glrlm_RunLengthNonUniformityNormalized	x	x	x	x

wavelet.HLL_glrlm_RunPercentage	x	x	x	x
wavelet.HLL_glrlm_RunVariance	x	x	x	x
wavelet.HLL_glrlm_ShortRunEmphasis	x	x	x	x
wavelet.HLL_glszm_GrayLevelNonUniformityNormalized	x	x	x	x
wavelet.HLL_glszm_GrayLevelVariance	x	x	x	x
wavelet.HLL_glszm_LargeAreaEmphasis	x	x	x	x
wavelet.HLL_glszm_SizeZoneNonUniformityNormalized	x	x	x	x
wavelet.HLL_glszm_SmallAreaEmphasis	x	x	x	x
wavelet.HLL_glszm_ZoneEntropy	x	x	x	
wavelet.HLL_glszm_ZonePercentage	x	x	x	x
wavelet.HLL_glszm_ZoneVariance	x	x	x	x
wavelet.HLL_gldm_DependenceEntropy	x	x		
wavelet.HLL_gldm_DependenceNonUniformityNormalized	x	x	x	x
wavelet.HLL_gldm_DependenceVariance	x	x	x	x
wavelet.HLL_gldm_GrayLevelVariance	x	x	x	x
wavelet.HLL_gldm_LargeDependenceEmphasis	x	x	x	x
wavelet.HLL_gldm_SmallDependenceEmphasis	x	x	x	x
wavelet.HLL_ngtdm_Complexity	x			
wavelet.HLL_ngtdm_Contrast	x		x	x
wavelet.HLH_firstorder_Entropy	x	x	x	x
wavelet.HLH_firstorder_10Percentile	x	x	x	x
wavelet.HLH_firstorder_90Percentile	x	x	x	x
wavelet.HLH_firstorder_Maximum	x	x	x	
wavelet.HLH_firstorder_InterquartileRange	x	x	x	x
wavelet.HLH_firstorder_MeanAbsoluteDeviation	x	x	x	x
wavelet.HLH_firstorder_RobustMeanAbsoluteDeviation	x	x	x	x
wavelet.HLH_firstorder_RootMeanSquared	x	x	x	x
wavelet.HLH_firstorder_Skewness	x		x	x
wavelet.HLH_firstorder_Variance	x	x	x	x
wavelet.HLH_firstorder_Uniformity	x	x	x	x
wavelet.HLH_glcm_ClusterProminence	x	x	x	x
wavelet.HLH_glcm_ClusterTendency	x	x	x	x
wavelet.HLH_glcm_Contrast	x	x	x	x
wavelet.HLH_glcm_DifferenceAverage	x	x	x	x
wavelet.HLH_glcm_DifferenceEntropy	x	x	x	x
wavelet.HLH_glcm_DifferenceVariance	x	x	x	x
wavelet.HLH_glcm_JointEnergy	x	x	x	x
wavelet.HLH_glcm_JointEntropy	x	x	x	x
wavelet.HLH_glcm_Imc2	x	x	x	
wavelet.HLH_glcm_Idm	x	x	x	x
wavelet.HLH_glcm_Id	x	x	x	x
wavelet.HLH_glcm_InverseVariance	x	x	x	x

	wavelet.HLH_glcm_MaximumProbability	x	x	x	x
	wavelet.HLH_glcm_SumEntropy	x	x	x	x
	wavelet.HLH_glcm_SumSquares	x	x	x	x
	wavelet.HLH_glrlm_GrayLevelNonUniformityNormalized	x	x	x	x
	wavelet.HLH_glrlm_GrayLevelVariance	x	x	x	x
	wavelet.HLH_glrlm_LongRunEmphasis	x	x	x	x
	wavelet.HLH_glrlm_RunEntropy	x	x	x	x
	wavelet.HLH_glrlm_RunLengthNonUniformityNormalized	x	x	x	x
	wavelet.HLH_glrlm_RunPercentage	x	x	x	x
	wavelet.HLH_glrlm_RunVariance	x	x	x	x
	wavelet.HLH_glrlm_ShortRunEmphasis	x	x	x	x
	wavelet.HLH_glszm_GrayLevelNonUniformityNormalized	x	x	x	x
	wavelet.HLH_glszm_GrayLevelVariance	x	x	x	x
	wavelet.HLH_glszm_LargeAreaEmphasis	x	x	x	x
	wavelet.HLH_glszm_SizeZoneNonUniformityNormalized	x	x	x	x
	wavelet.HLH_glszm_SmallAreaEmphasis	x	x	x	x
	wavelet.HLH_glszm_ZoneEntropy	x	x	x	x
	wavelet.HLH_glszm_ZonePercentage	x	x	x	x
	wavelet.HLH_glszm_ZoneVariance	x	x	x	x
	wavelet.HLH_gldm_DependenceEntropy	x	x	x	x
	wavelet.HLH_gldm_DependenceNonUniformityNormalized	x	x	x	x
	wavelet.HLH_gldm_DependenceVariance	x	x	x	x
	wavelet.HLH_gldm_GrayLevelVariance	x	x	x	x
	wavelet.HLH_gldm_LargeDependenceEmphasis	x	x	x	x
	wavelet.HLH_gldm_SmallDependenceEmphasis	x	x	x	x
	wavelet.HLH_ngtdm_Complexity	x	x	x	x
	wavelet.HLH_ngtdm_Contrast	x	x	x	
	wavelet.HLH_ngtdm_Strength	x	x	x	
	wavelet.HHL_firstorder_Entropy	x	x	x	x
	wavelet.HHL_firstorder_10Percentile	x	x	x	x
	wavelet.HHL_firstorder_90Percentile	x	x	x	x
	wavelet.HHL_firstorder_InterquartileRange	x	x	x	x
	wavelet.HHL_firstorder_MeanAbsoluteDeviation	x	x	x	x
	wavelet.HHL_firstorder_RobustMeanAbsoluteDeviation	x	x	x	x
	wavelet.HHL_firstorder_RootMeanSquared	x	x	x	
	wavelet.HHL_firstorder_Variance	x	x	x	x
	wavelet.HHL_firstorder_Uniformity	x	x	x	x
	wavelet.HHL_glcm_ClusterProminence	x	x	x	x
	wavelet.HHL_glcm_ClusterTendency	x	x	x	x
	wavelet.HHL_glcm_Contrast	x	x	x	x

	wavelet.HHL_glcm_DifferenceAverage	x	x	x	x
	wavelet.HHL_glcm_DifferenceEntropy	x	x	x	x
	wavelet.HHL_glcm_DifferenceVariance	x	x	x	x
	wavelet.HHL_glcm_JointEnergy	x	x	x	x
	wavelet.HHL_glcm_JointEntropy	x	x	x	x
	wavelet.HHL_glcm_Imc2	x	x	x	
	wavelet.HHL_glcm_Idm	x	x	x	x
	wavelet.HHL_glcm_Id	x	x	x	x
	wavelet.HHL_glcm_InverseVariance	x	x	x	x
	wavelet.HHL_glcm_MaximumProbability	x	x	x	x
	wavelet.HHL_glcm_SumEntropy	x	x	x	x
	wavelet.HHL_glcm_SumSquares	x	x	x	x
	wavelet.HHL_glrlm_GrayLevelNonUniformityNormalized	x	x	x	x
	wavelet.HHL_glrlm_GrayLevelVariance	x	x	x	x
	wavelet.HHL_glrlm_LongRunEmphasis	x	x	x	x
	wavelet.HHL_glrlm_RunEntropy	x	x	x	x
	wavelet.HHL_glrlm_RunLengthNonUniformityNormalized	x	x	x	x
	wavelet.HHL_glrlm_RunPercentage	x	x	x	x
	wavelet.HHL_glrlm_RunVariance	x	x	x	x
	wavelet.HHL_glrlm_ShortRunEmphasis	x	x	x	x
	wavelet.HHL_glszm_GrayLevelNonUniformityNormalized	x	x	x	x
	wavelet.HHL_glszm_GrayLevelVariance	x	x	x	x
	wavelet.HHL_glszm_LargeAreaEmphasis	x	x	x	x
	wavelet.HHL_glszm_SizeZoneNonUniformityNormalized	x	x	x	x
	wavelet.HHL_glszm_SmallAreaEmphasis	x	x	x	x
	wavelet.HHL_glszm_ZonePercentage	x	x	x	x
	wavelet.HHL_glszm_ZoneVariance	x	x	x	x
	wavelet.HHL_gldm_DependenceEntropy	x	x	x	x
	wavelet.HHL_gldm_DependenceNonUniformityNormalized	x	x	x	x
	wavelet.HHL_gldm_DependenceVariance	x	x	x	x
	wavelet.HHL_gldm_GrayLevelVariance	x	x	x	x
	wavelet.HHL_gldm_LargeDependenceEmphasis	x	x	x	x
	wavelet.HHL_gldm_SmallDependenceEmphasis	x	x	x	x
	wavelet.HHL_gldm_SmallDependenceHighGrayLevelEmphasis	x	x		
	wavelet.HHH_firstorder_Entropy	x	x	x	x
	wavelet.HHH_firstorder_10Percentile	x	x	x	x
	wavelet.HHH_firstorder_90Percentile	x	x	x	x
	wavelet.HHH_firstorder_InterquartileRange	x	x	x	x
	wavelet.HHH_firstorder_MeanAbsoluteDeviation	x	x	x	x

	wavelet.HHH_firstorder_RobustMeanAbsoluteDeviation	x	x	x	x
	wavelet.HHH_firstorder_RootMeanSquared	x	x	x	x
	wavelet.HHH_firstorder_Kurtosis	x	x	x	x
	wavelet.HHH_firstorder_Variance	x	x	x	x
	wavelet.HHH_firstorder_Uniformity	x	x	x	x
	wavelet.HHH_glcm_ClusterProminence	x	x	x	x
	wavelet.HHH_glcm_ClusterTendency	x	x	x	x
	wavelet.HHH_glcm_Contrast	x	x	x	x
	wavelet.HHH_glcm_DifferenceAverage	x	x	x	x
	wavelet.HHH_glcm_DifferenceEntropy	x	x	x	x
	wavelet.HHH_glcm_DifferenceVariance	x	x	x	x
	wavelet.HHH_glcm_JointEnergy	x	x	x	x
	wavelet.HHH_glcm_JointEntropy	x	x	x	x
	wavelet.HHH_glcm_Imc2	x	x	x	
	wavelet.HHH_glcm_Idm	x	x	x	x
	wavelet.HHH_glcm_Id	x	x	x	x
	wavelet.HHH_glcm_InverseVariance	x	x	x	x
	wavelet.HHH_glcm_MaximumProbability	x	x	x	x
	wavelet.HHH_glcm_SumEntropy	x	x	x	x
	wavelet.HHH_glcm_SumSquares	x	x	x	x
	wavelet.HHH_glrlm_GrayLevelNonUniformityNormalized	x	x	x	x
	wavelet.HHH_glrlm_GrayLevelVariance	x	x	x	x
	wavelet.HHH_glrlm_LongRunEmphasis	x	x	x	x
	wavelet.HHH_glrlm_RunEntropy	x	x	x	x
	wavelet.HHH_glrlm_RunLengthNonUniformityNormalized	x	x	x	x
	wavelet.HHH_glrlm_RunPercentage	x	x	x	x
	wavelet.HHH_glrlm_RunVariance	x	x	x	x
	wavelet.HHH_glrlm_ShortRunEmphasis	x	x	x	x
	wavelet.HHH_glszm_GrayLevelNonUniformityNormalized	x	x	x	x
	wavelet.HHH_glszm_GrayLevelVariance	x	x	x	x
	wavelet.HHH_glszm_LargeAreaEmphasis	x	x	x	x
	wavelet.HHH_glszm_LargeAreaLowGrayLevelEmphasis	x	x	x	
	wavelet.HHH_glszm_SizeZoneNonUniformityNormalized	x	x	x	x
	wavelet.HHH_glszm_SizeZoneNonUniformityNormalized	x	x	x	x
	wavelet.HHH_glszm_SmallAreaEmphasis	x	x	x	x
	wavelet.HHH_glszm_ZonePercentage	x	x	x	x
	wavelet.HHH_glszm_ZoneVariance	x	x	x	x
	wavelet.HHH_gldm_DependenceEntropy	x	x	x	x
	wavelet.HHH_gldm_DependenceNonUniformityNormalized	x	x	x	x
	wavelet.HHH_gldm_DependenceVariance	x	x	x	x
	wavelet.HHH_gldm_GrayLevelVariance	x	x	x	x

wavelet.HHH_gldm_LargeDependenceEmphasis	x	x	x	x
wavelet.HHH_gldm_SmallDependenceEmphasis	x	x	x	x
wavelet.HHH_ngtdm_Complexity	x	x	x	x
wavelet.HHH_ngtdm_Contrast	x		x	
wavelet.HHH_ngtdm_Strength	x	x	x	x
wavelet.LLL_firstorder_Minimum	x	x	x	x
wavelet.LLL_firstorder_10Percentile	x	x	x	x
wavelet.LLL_firstorder_90Percentile	x	x	x	x
wavelet.LLL_firstorder_Mean	x	x	x	x
wavelet.LLL_firstorder_Median	x	x	x	x
wavelet.LLL_firstorder_RootMeanSquared	x	x	x	x
wavelet.LLL_glrlm_GrayLevelNonUniformity	x	x	x	x
wavelet.LLL_glrlm_LongRunEmphasis	x	x		x
wavelet.LLL_glrlm_RunLengthNonUniformityNormalized	x	x		x
wavelet.LLL_glrlm_RunPercentage	x	x		x
wavelet.LLL_glrlm_RunVariance	x	x		x
wavelet.LLL_glrlm_ShortRunEmphasis	x	x		x
wavelet.LLL_glszm_GrayLevelNonUniformity	x	x	x	x
wavelet.LLL_glszm_LargeAreaEmphasis	x	x		x
wavelet.LLL_glszm_SmallAreaEmphasis	x	x		x
wavelet.LLL_glszm_ZonePercentage	x	x		x
wavelet.LLL_glszm_ZoneVariance	x	x	x	x
wavelet.LLL_gldm_DependenceNonUniformityNormalized	x	x		x
wavelet.LLL_gldm_DependenceVariance	x	x	x	x
wavelet.LLL_gldm_GrayLevelNonUniformity	x	x	x	x
wavelet.LLL_gldm_LargeDependenceEmphasis	x	x		x
wavelet.LLL_gldm_SmallDependenceEmphasis	x	x		x
wavelet.LLL_ngtdm_Coarseness	x	x		

Table S2. List of robust and reliable radiomics features by feature class. 314 features were identified as robust to interobserver and inter-software differences. This includes 22 original (unfiltered) and 292 wavelet (filtered) features with 70 first order, 88 GLCM, 59 GLRLM, 54 GLSZM, 40 GLDM and 3 NGTDM features.

First Order	GLCM	GLRLM	GLSZM	GLDM	NGTDM
original_firstorder_Minimum	original_glcm_Idm	original_glrlm_GrayLevelNonUniformity	original_glszm_GrayLevelNonUniformity	original_gldm_DependenceNonUniformityNormalized	wavelet.HLH_ngtdm_Complexity
original_firstorder_10Percentile	original_glcm_Id	original_glrlm_LongRunEmphasis	original_glszm_LargeAreaEmphasis	original_gldm_DependenceVariance	wavelet.HHH_ngtdm_Complexity
original_firstorder_90Percentile	original_glcm_InverseVariance	original_glrlm_RunLengthNonUniformityNormalized	original_glszm_ZonePercentage	original_gldm_GrayLevelNonUniformity	wavelet.HHH_ngtdm_Strength
original_firstorder_Mean	wavelet.LLH_glcm_Contrast	original_glrlm_RunPercentage	original_glszm_ZoneVariance	wavelet.LLH_gldm_DependenceNonUniformityNormalized	

original_firstorder_Median	wavelet.LLH_glcm_DifferenceAverage	original_glrlm_RunVariance	wavelet.LLH_glszm_GrayLevelNonUniformityNormalized	wavelet.LLH_gldm_DependenceVariance
original_firstorder_RootMeanSquared	wavelet.LLH_glcm_DifferenceEntropy	original_glrlm_ShortRunEmphasis	wavelet.LLH_glszm_LargeAreaEmphasis	wavelet.LLH_gldm_LargeDependenceEmphasis
wavelet.LLH_firstorder_Entropy	wavelet.LLH_glcm_DifferenceVariance	wavelet.LLH_glrlm_GrayLevelNonUniformityNormalized	wavelet.LLH_glszm_SizeZoneNonUniformityNormalized	wavelet.LLH_gldm_SmallDependenceEmphasis
wavelet.LLH_firstorder_10Percentile	wavelet.LLH_glcm_JointEnergy	wavelet.LLH_glrlm_LongRunEmphasis	wavelet.LLH_glszm_SmallAreaEmphasis	wavelet.LHL_gldm_DependenceNonUniformityNormalized
wavelet.LLH_firstorder_90Percentile	wavelet.LLH_glcm_JointEntropy	wavelet.LLH_glrlm_RunLengthNonUniformityNormalized	wavelet.LLH_glszm_ZonePercentage	wavelet.LHL_gldm_DependenceVariance
wavelet.LLH_firstorder_InterquartileRange	wavelet.LLH_glcm_Idm	wavelet.LLH_glrlm_RunPercentage	wavelet.LLH_glszm_ZoneVariance	wavelet.LHL_gldm_LargeDependenceEmphasis
wavelet.LLH_firstorder_MeanAbsoluteDeviation	wavelet.LLH_glcm_Id	wavelet.LLH_glrlm_RunVariance	wavelet.LHL_glszm_GrayLevelNonUniformityNormalized	wavelet.LHL_gldm_SmallDependenceEmphasis
wavelet.LLH_firstorder_RobustMeanAbsoluteDeviation	wavelet.LLH_glcm_InverseVariance	wavelet.LLH_glrlm_ShortRunEmphasis	wavelet.LHL_glszm_LargeAreaEmphasis	wavelet.LHH_gldm_DependenceNonUniformityNormalized
wavelet.LLH_firstorder_Uniformity	wavelet.LLH_glcm_MaximumProbability	wavelet.LHL_glrlm_GrayLevelNonUniformityNormalized	wavelet.LHL_glszm_SizeZoneNonUniformityNormalized	wavelet.LHH_gldm_DependenceVariance
wavelet.LHL_firstorder_Entropy	wavelet.LHL_glcm_DifferenceAverage	wavelet.LHL_glrlm_LongRunEmphasis	wavelet.LHL_glszm_SmallAreaEmphasis	wavelet.LHH_gldm_LargeDependenceEmphasis
wavelet.LHL_firstorder_90Percentile	wavelet.LHL_glcm_DifferenceEntropy	wavelet.LHL_glrlm_RunEntropy	wavelet.LHL_glszm_ZonePercentage	wavelet.LHH_gldm_SmallDependenceEmphasis
wavelet.LHL_firstorder_Maximum	wavelet.LHL_glcm_JointEnergy	wavelet.LHL_glrlm_RunLengthNonUniformityNormalized	wavelet.LHL_glszm_ZoneVariance	wavelet.HLL_gldm_DependenceNonUniformityNormalized
wavelet.LHL_firstorder_InterquartileRange	wavelet.LHL_glcm_JointEntropy	wavelet.LHL_glrlm_RunPercentage	wavelet.LHH_glszm_GrayLevelNonUniformityNormalized	wavelet.HLL_gldm_DependenceVariance
wavelet.LHL_firstorder_MeanAbsoluteDeviation	wavelet.LHL_glcm_Idm	wavelet.LHL_glrlm_RunVariance	wavelet.LHH_glszm_LargeAreaEmphasis	wavelet.HLL_gldm_GrayLevelVariance
wavelet.LHL_firstorder_RobustMeanAbsoluteDeviation	wavelet.LHL_glcm_Id	wavelet.LHL_glrlm_ShortRunEmphasis	wavelet.LHH_glszm_SizeZoneNonUniformityNormalized	wavelet.HLL_gldm_LargeDependenceEmphasis
wavelet.LHL_firstorder_RootMeanSquared	wavelet.LHL_glcm_InverseVariance	wavelet.LHH_glrlm_GrayLevelNonUniformityNormalized	wavelet.LHH_glszm_SmallAreaEmphasis	wavelet.HLL_gldm_SmallDependenceEmphasis
wavelet.LHL_firstorder_Uniformity	wavelet.LHL_glcm_MaximumProbability	wavelet.LHH_glrlm_LongRunEmphasis	wavelet.LHH_glszm_ZonePercentage	wavelet.HLH_gldm_DependenceEntropy
wavelet.LHH_firstorder_Entropy	wavelet.LHL_glcm_SumEntropy	wavelet.LHH_glrlm_RunEntropy	wavelet.LHH_glszm_ZoneVariance	wavelet.HLH_gldm_DependenceNonUniformityNormalized

wavelet.LHH_firstorder_10Percentile	wavelet.LHH_glcm_ClusterTendency	wavelet.LHH_glrlm_RunLengthNonUniformityNormalized	wavelet.HLL_glszm_GrayLevelNonUniformityNormalized	wavelet.HLH_gldm_DependenceVariance
wavelet.LHH_firstorder_90Percentile	wavelet.LHH_glcm_Contrast	wavelet.LHH_glrlm_RunPercentage	wavelet.HLL_glszm_GrayLevelVariance	wavelet.HLH_gldm_GrayLevelVariance
wavelet.LHH_firstorder_InterquartileRange	wavelet.LHH_glcm_DifferenceAverage	wavelet.LHH_glrlm_RunVariance	wavelet.HLL_glszm_LargeAreaEmphasis	wavelet.HLH_gldm_LargeDependenceEmphasis
wavelet.LHH_firstorder_MeanAbsoluteDeviation	wavelet.LHH_glcm_DifferenceEntropy	wavelet.LHH_glrlm_ShortRunEmphasis	wavelet.HLL_glszm_SizeZoneNonUniformityNormalized	wavelet.HLH_gldm_SmallDependenceEmphasis
wavelet.LHH_firstorder_RobustMeanAbsoluteDeviation	wavelet.LHH_glcm_JointEnergy	wavelet.HLL_glrlm_GrayLevelNonUniformityNormalized	wavelet.HLL_glszm_SmallAreaEmphasis	wavelet.HHL_gldm_DependenceEntropy
wavelet.LHH_firstorder_Uniformity	wavelet.LHH_glcm_JointEntropy	wavelet.HLL_glrlm_GrayLevelVariance	wavelet.HLL_glszm_ZonePercentage	wavelet.HHL_gldm_DependenceNonUniformityNormalized
wavelet.HLL_firstorder_Entropy	wavelet.LHH_glcm_Idm	wavelet.HLL_glrlm_LongRunEmphasis	wavelet.HLL_glszm_ZoneVariance	wavelet.HHL_gldm_DependenceVariance
wavelet.HLL_firstorder_10Percentile	wavelet.LHH_glcm_Id	wavelet.HLL_glrlm_RunEntropy	wavelet.HLH_glszm_GrayLevelNonUniformityNormalized	wavelet.HHL_gldm_GrayLevelVariance
wavelet.HLL_firstorder_90Percentile	wavelet.LHH_glcm_InverseVariance	wavelet.HLL_glrlm_RunLengthNonUniformityNormalized	wavelet.HLH_glszm_GrayLevelVariance	wavelet.HHL_gldm_LargeDependenceEmphasis
wavelet.HLL_firstorder_InterquartileRange	wavelet.LHH_glcm_MaximumProbability	wavelet.HLL_glrlm_RunPercentage	wavelet.HLH_glszm_LargeAreaEmphasis	wavelet.HHL_gldm_SmallDependenceEmphasis
wavelet.HLL_firstorder_MeanAbsoluteDeviation	wavelet.LHH_glcm_SumEntropy	wavelet.HLL_glrlm_RunVariance	wavelet.HLH_glszm_SizeZoneNonUniformityNormalized	wavelet.HHH_gldm_DependenceEntropy
wavelet.HLL_firstorder_RobustMeanAbsoluteDeviation	wavelet.LHH_glcm_SumSquares	wavelet.HLL_glrlm_ShortRunEmphasis	wavelet.HLH_glszm_SmallAreaEmphasis	wavelet.HHH_gldm_DependenceNonUniformityNormalized
wavelet.HLL_firstorder_RootMeanSquared	wavelet.HLL_glcm_ClusterTendency	wavelet.HLH_glrlm_GrayLevelNonUniformityNormalized	wavelet.HLH_glszm_ZoneEntropy	wavelet.HHH_gldm_DependenceVariance
wavelet.HLL_firstorder_Variance	wavelet.HLL_glcm_Contrast	wavelet.HLH_glrlm_GrayLevelVariance	wavelet.HLH_glszm_ZonePercentage	wavelet.HHH_gldm_GrayLevelVariance
wavelet.HLL_firstorder_Uniformity	wavelet.HLL_glcm_DifferenceAverage	wavelet.HLH_glrlm_LongRunEmphasis	wavelet.HLH_glszm_ZoneVariance	wavelet.HHH_gldm_LargeDependenceEmphasis
wavelet.HLH_firstorder_Entropy	wavelet.HLL_glcm_DifferenceEntropy	wavelet.HLH_glrlm_RunEntropy	wavelet.HHL_glszm_GrayLevelNonUniformityNormalized	wavelet.HHH_gldm_SmallDependenceEmphasis
wavelet.HLH_firstorder_10Percentile	wavelet.HLL_glcm_DifferenceVariance	wavelet.HLH_glrlm_RunLengthNonUniformityNormalized	wavelet.HHL_glszm_GrayLevelVariance	wavelet.LLL_gldm_DependenceVariance
wavelet.HLH_firstorder_90Percentile	wavelet.HLL_glcm_JointEnergy	wavelet.HLH_glrlm_RunPercentage	wavelet.HHL_glszm_LargeAreaEmphasis	wavelet.LLL_gldm_GrayLevelNonUniformity

wavelet.HLH_firstorder_InterquartileRange	wavelet.HLL_glcm_JointEntropy	wavelet.HLH_glrlm_RunVariance	wavelet.HHL_glszm_SizeZoneNonUniformityNormalized
wavelet.HLH_firstorder_MeanAbsoluteDeviation	wavelet.HLL_glcm_Idm	wavelet.HLH_glrlm_ShortRunEmphasis	wavelet.HHL_glszm_SmallAreaEmphasis
wavelet.HLH_firstorder_RobustMeanAbsoluteDeviation	wavelet.HLL_glcm_Id	wavelet.HHL_glrlm_GrayLevelNonUniformityNormalized	wavelet.HHL_glszm_ZonePercentage
wavelet.HLH_firstorder_RootMeanSquared	wavelet.HLL_glcm_InverseVariance	wavelet.HHL_glrlm_GrayLevelVariance	wavelet.HHL_glszm_ZoneVariance
wavelet.HLH_firstorder_Variance	wavelet.HLL_glcm_SumEntropy	wavelet.HHL_glrlm_LongRunEmphasis	wavelet.HHH_glszm_GrayLevelNonUniformityNormalized
wavelet.HLH_firstorder_Uniformity	wavelet.HLL_glcm_SumSquares	wavelet.HHL_glrlm_RunEntropy	wavelet.HHH_glszm_GrayLevelVariance
wavelet.HHL_firstorder_Entropy	wavelet.HLH_glcm_ClusterProminence	wavelet.HHL_glrlm_RunLengthNonUniformityNormalized	wavelet.HHH_glszm_LargeAreaEmphasis
wavelet.HHL_firstorder_10Percentile	wavelet.HLH_glcm_ClusterTendency	wavelet.HHL_glrlm_RunPercentage	wavelet.HHL_glszm_SizeZoneNonUniformityNormalized
wavelet.HHL_firstorder_90Percentile	wavelet.HLH_glcm_Contrast	wavelet.HHL_glrlm_RunVariance	wavelet.HHL_glszm_SmallAreaEmphasis
wavelet.HHL_firstorder_InterquartileRange	wavelet.HLH_glcm_DifferenceAverage	wavelet.HHL_glrlm_ShortRunEmphasis	wavelet.HHL_glszm_ZonePercentage
wavelet.HHL_firstorder_MeanAbsoluteDeviation	wavelet.HLH_glcm_DifferenceEntropy	wavelet.HHH_glrlm_GrayLevelNonUniformityNormalized	wavelet.HHL_glszm_ZoneVariance
wavelet.HHL_firstorder_RobustMeanAbsoluteDeviation	wavelet.HLH_glcm_DifferenceVariance	wavelet.HHH_glrlm_GrayLevelVariance	wavelet.HHH_glszm_GrayLevelNonUniformityNormalized
wavelet.HHL_firstorder_Variance	wavelet.HLH_glcm_JointEnergy	wavelet.HHH_glrlm_LongRunEmphasis	wavelet.HHH_glszm_GrayLevelVariance
wavelet.HHL_firstorder_Uniformity	wavelet.HLH_glcm_JointEntropy	wavelet.HHH_glrlm_RunEntropy	wavelet.HHH_glszm_LargeAreaEmphasis
wavelet.HHH_firstorder_Entropy	wavelet.HLH_glcm_Idm	wavelet.HHH_glrlm_RunLengthNonUniformityNormalized	wavelet.HHH_glszm_SizeZoneNonUniformityNormalized
wavelet.HHH_firstorder_10Percentile	wavelet.HLH_glcm_Id	wavelet.HHH_glrlm_RunPercentage	wavelet.HHH_glszm_SizeZoneNonUniformityNormalized
wavelet.HHH_firstorder_90Percentile	wavelet.HLH_glcm_InverseVariance	wavelet.HHH_glrlm_RunVariance	wavelet.HHH_glszm_SmallAreaEmphasis
wavelet.HHH_firstorder_InterquartileRange	wavelet.HLH_glcm_MaximumProbability	wavelet.HHH_glrlm_ShortRunEmphasis	wavelet.HHH_glszm_ZonePercentage

wavelet.HHH_firstorder_MeanAbsoluteDeviation	wavelet.HLH_glcm_SumEntropy	wavelet.LLL_glrlm_GrayLevelNonUniformity	wavelet.HHH_glszm_ZoneVariance
wavelet.HHH_firstorder_RobustMeanAbsoluteDeviation	wavelet.HLH_glcm_SumSquares		wavelet.LLL_glszm_GrayLevelNonUniformity
wavelet.HHH_firstorder_RootMeanSquared	wavelet.HHL_glcm_ClusterProminence		wavelet.LLL_glszm_ZoneVariance
wavelet.HHH_firstorder_Kurtosis	wavelet.HHL_glcm_ClusterTendency		
wavelet.HHH_firstorder_Variance	wavelet.HHL_glcm_Contrast		
wavelet.HHH_firstorder_Uniformity	wavelet.HHL_glcm_DifferenceAverage		
wavelet.LLL_firstorder_Minimum	wavelet.HHL_glcm_DifferenceEntropy		
wavelet.LLL_firstorder_10Percentile	wavelet.HHL_glcm_DifferenceVariance		
wavelet.LLL_firstorder_90Percentile	wavelet.HHL_glcm_JointEnergy		
wavelet.LLL_firstorder_Mean	wavelet.HHL_glcm_JointEntropy		
wavelet.LLL_firstorder_Median	wavelet.HHL_glcm_Idm		
wavelet.LLL_firstorder_RootMeanSquared	wavelet.HHL_glcm_Id		
	wavelet.HHL_glcm_InverseVariance		
	wavelet.HHL_glcm_MaximumProbability		
	wavelet.HHL_glcm_SumEntropy		
	wavelet.HHL_glcm_SumSquares		
	wavelet.HHH_glcm_ClusterProminence		
	wavelet.HHH_glcm_ClusterTendency		
	wavelet.HHH_glcm_Contrast		
	wavelet.HHH_glcm_DifferenceAverage		

wavelet.HHH_glcm_DifferenceEntropy
wavelet.HHH_glcm_DifferenceVariance
wavelet.HHH_glcm_JointEnergy
wavelet.HHH_glcm_JointEntropy
wavelet.HHH_glcm_Idm
wavelet.HHH_glcm_Id
wavelet.HHH_glcm_InverseVariance
wavelet.HHH_glcm_MaximumProbability
wavelet.HHH_glcm_SumEntropy
wavelet.HHH_glcm_SumSquares

Table S3. Comparison of preclinical and clinical interobserver delineation variations in radiomics analysis. Clinical contours were for 3 different tumour models including: non-small cell lung cancer (NSCLC), head and neck squamous cell carcinoma (HNSCC) and malignant pleural mesothelioma (MPM). Features determined as reliable with an ICC >0.8 are marked with an 'x'.

	**Preclinical lungs**	**Clinical NSCLC**	**Clinical HNSCC**	**Clinical MPM**
original_shape_LeastAxisLength	x	x	x	
original_firstorder_10Percentile	x	x		
original_firstorder_90Percentile	x	x	x	
original_firstorder_Mean	x	x	x	
original_firstorder_Median	x	x	x	x
original_firstorder_RootMeanSquared	x	x	x	
original_glcm_DifferenceAverage	x	x	x	
original_glcm_DifferenceEntropy	x	x	x	x
original_glcm_InverseVariance	x	x	x	
original_glrlm_GrayLevelNonUniformity	x	x	x	x
original_glrlm_LongRunEmphasis	x	x	x	
original_glrlm_RunPercentage	x	x	x	
original_glrlm_ShortRunEmphasis	x	x	x	
original_glszm_GrayLevelNonUniformity	x	x	x	x
original_glszm_SizeZoneNonUniformityNormalized	x	x		
original_glszm_ZonePercentage	x	x	x	
original_glszm_ZoneVariance	x	x	x	
original_gldm_GrayLevelNonUniformity	x	x	x	x

wavelet.LLH_firstorder_Entropy	x	x	x	x
wavelet.LLH_firstorder_10Percentile	x	x		
wavelet.LLH_firstorder_90Percentile	x			x
wavelet.LLH_firstorder_InterquartileRange	x	x	x	x
wavelet.LLH_firstorder_MeanAbsoluteDeviation	x	x		x
wavelet.LLH_firstorder_RobustMeanAbsoluteDeviation	x	x		x
wavelet.LLH_firstorder_RootMeanSquared	x	x		x
wavelet.LLH_firstorder_Variance	x	x		x
wavelet.LLH_firstorder_Uniformity	x	x	x	
wavelet.LLH_glcm_ClusterTendency	x	x		x
wavelet.LLH_glcm_Contrast	x	x		x
wavelet.LLH_glcm_DifferenceAverage	x	x		x
wavelet.LLH_glcm_DifferenceEntropy	x	x	x	x
wavelet.LLH_glcm_DifferenceVariance	x	x		x
wavelet.LLH_glcm_Imc1	x	x	x	
wavelet.LLH_glcm_Imc2	x	x	x	
wavelet.LLH_glcm_InverseVariance	x	x	x	
wavelet.LLH_glcm_SumEntropy	x	x	x	x
wavelet.LLH_glrlm_GrayLevelNonUniformityNormalized	x	x	x	
wavelet.LLH_glrlm_GrayLevelVariance	x	x		x
wavelet.LLH_glrlm_LongRunEmphasis	x	x	x	
wavelet.LLH_glrlm_RunEntropy	x			x
wavelet.LLH_glrlm_RunPercentage	x	x	x	
wavelet.LLH_glrlm_RunVariance	x			x
wavelet.LLH_glrlm_ShortRunEmphasis	x	x	x	
wavelet.LLH_glszm_GrayLevelNonUniformityNormalized	x			x
wavelet.LLH_glszm_GrayLevelVariance	x	x		x
wavelet.LLH_glszm_LargeAreaEmphasis	x	x	x	
wavelet.LLH_glszm_SizeZoneNonUniformityNormalized	x	x		
wavelet.LLH_glszm_SmallAreaEmphasis	x	x		
wavelet.LLH_glszm_ZonePercentage	x	x	x	
wavelet.LLH_glszm_ZoneVariance	x	x	x	
wavelet.LLH_gldm_GrayLevelVariance	x	x		x
wavelet.LLH_gldm_SmallDependenceEmphasis	x	x		
wavelet.LHL_firstorder_Entropy	x	x	x	x
wavelet.LHL_firstorder_10Percentile	x	x		x
wavelet.LHL_firstorder_90Percentile	x	x	x	x
wavelet.LHL_firstorder_InterquartileRange	x	x	x	x
wavelet.LHL_firstorder_MeanAbsoluteDeviation	x	x	x	x
wavelet.LHL_firstorder_RobustMeanAbsoluteDeviation	x	x	x	x
wavelet.LHL_firstorder_RootMeanSquared	x	x		x
wavelet.LHL_firstorder_Variance	x	x		x

wavelet.LHL_firstorder_Uniformity	x	x	x	x
wavelet.LHL_glcm_ClusterTendency	x	x	x	x
wavelet.LHL_glcm_Contrast	x	x	x	x
wavelet.LHL_glcm_Correlation	x	x		
wavelet.LHL_glcm_DifferenceAverage	x	x	x	x
wavelet.LHL_glcm_DifferenceEntropy	x	x	x	x
wavelet.LHL_glcm_DifferenceVariance	x	x		x
wavelet.LHL_glcm_Imc1	x	x	x	
wavelet.LHL_glcm_InverseVariance	x	x	x	
wavelet.LHL_glcm_SumEntropy	x	x	x	x
wavelet.LHL_glrlm_GrayLevelNonUniformity	x	x	x	x
wavelet.LHL_glrlm_GrayLevelNonUniformityNormalized	x	x	x	x
wavelet.LHL_glrlm_GrayLevelVariance	x	x		x
wavelet.LHL_glrlm_LongRunEmphasis	x	x	x	
wavelet.LHL_glrlm_RunEntropy	x	x	x	x
wavelet.LHL_glrlm_RunPercentage	x	x	x	
wavelet.LHL_glrlm_RunVariance	x	x	x	
wavelet.LHL_glrlm_ShortRunEmphasis	x	x	x	
wavelet.LHL_glszm_GrayLevelNonUniformityNormalized	x	x	x	x
wavelet.LHL_glszm_GrayLevelVariance	x	x		x
wavelet.LHL_glszm_LargeAreaEmphasis	x	x	x	
wavelet.LHL_glszm_ZonePercentage	x	x	x	
wavelet.LHL_glszm_ZoneVariance	x	x	x	
wavelet.LHL_gldm_GrayLevelNonUniformity	x	x	x	x
wavelet.LHL_gldm_GrayLevelVariance	x	x		x
wavelet.LHL_gldm_LargeDependenceEmphasis	x	x	x	
wavelet.LHL_gldm_SmallDependenceEmphasis	x	x	x	
wavelet.LHL_ngtdm_Contrast	x	x	x	
wavelet.LHH_firstorder_Entropy	x	x	x	x
wavelet.LHH_firstorder_10Percentile	x	x	x	x
wavelet.LHH_firstorder_90Percentile	x	x	x	x
wavelet.LHH_firstorder_InterquartileRange	x	x	x	x
wavelet.LHH_firstorder_MeanAbsoluteDeviation	x	x	x	x
wavelet.LHH_firstorder_RobustMeanAbsoluteDeviation	x	x	x	x
wavelet.LHH_firstorder_Variance	x	x	x	x
wavelet.LHH_firstorder_Uniformity	x	x	x	
wavelet.LHH_glcm_ClusterTendency	x	x	x	x
wavelet.LHH_glcm_Contrast	x	x	x	x
wavelet.LHH_glcm_DifferenceAverage	x	x	x	x
wavelet.LHH_glcm_DifferenceEntropy	x	x	x	x
wavelet.LHH_glcm_DifferenceVariance	x	x	x	x
wavelet.LHH_glcm_InverseVariance	x	x	x	x
wavelet.LHH_glcm_SumEntropy	x	x	x	x

wavelet.LHH_glrlm_GrayLevelNonUniformityNormalized	x	x	x	x
wavelet.LHH_glrlm_GrayLevelVariance	x	x		x
wavelet.LHH_glrlm_LongRunEmphasis	x	x	x	
wavelet.LHH_glrlm_RunEntropy	x	x		x
wavelet.LHH_glrlm_RunPercentage	x	x	x	
wavelet.LHH_glrlm_RunVariance	x	x		x
wavelet.LHH_glrlm_ShortRunEmphasis	x	x	x	
wavelet.LHH_glszm_GrayLevelNonUniformityNormalized	x	x		
wavelet.LHH_glszm_GrayLevelVariance	x	x		x
wavelet.LHH_glszm_LargeAreaEmphasis	x	x	x	
wavelet.LHH_glszm_ZonePercentage	x	x	x	
wavelet.LHH_gldm_GrayLevelVariance	x	x		x
wavelet.LHH_gldm_LargeDependenceEmphasis	x	x		
wavelet.HLL_firstorder_Entropy	x	x	x	x
wavelet.HLL_firstorder_10Percentile	x	x	x	
wavelet.HLL_firstorder_90Percentile	x	x	x	
wavelet.HLL_firstorder_InterquartileRange	x	x	x	
wavelet.HLL_firstorder_MeanAbsoluteDeviation	x	x	x	
wavelet.HLL_firstorder_RobustMeanAbsoluteDeviation	x	x	x	
wavelet.HLL_firstorder_RootMeanSquared	x	x	x	
wavelet.HLL_firstorder_Variance	x	x	x	
wavelet.HLL_firstorder_Uniformity	x	x	x	
wavelet.HLL_glcm_ClusterProminence	x	x		
wavelet.HLL_glcm_ClusterTendency	x	x	x	
wavelet.HLL_glcm_Contrast	x	x	x	
wavelet.HLL_glcm_DifferenceAverage	x	x	x	
wavelet.HLL_glcm_DifferenceEntropy	x	x	x	x
wavelet.HLL_glcm_DifferenceVariance	x	x	x	
wavelet.HLL_glcm_Imc1	x	x	x	
wavelet.HLL_glcm_Imc2	x	x	x	x
wavelet.HLL_glcm_InverseVariance	x	x	x	
wavelet.HLL_glcm_SumEntropy	x	x	x	x
wavelet.HLL_glrlm_GrayLevelNonUniformityNormalized	x	x	x	
wavelet.HLL_glrlm_GrayLevelVariance	x	x	x	
wavelet.HLL_glrlm_LongRunEmphasis	x	x	x	
wavelet.HLL_glrlm_RunEntropy	x	x	x	x
wavelet.HLL_glrlm_RunPercentage	x	x	x	
wavelet.HLL_glrlm_RunVariance	x	x	x	
wavelet.HLL_glrlm_ShortRunEmphasis	x	x	x	
wavelet.HLL_glszm_GrayLevelNonUniformityNormalized	x	x	x	x
wavelet.HLL_glszm_GrayLevelVariance	x	x	x	
wavelet.HLL_glszm_LargeAreaEmphasis	x	x	x	

wavelet.HLL_glszm_ZoneEntropy	x	x	x	x
wavelet.HLL_glszm_ZonePercentage	x	x	x	
wavelet.HLL_glszm_ZoneVariance	x	x	x	
wavelet.HLL_gldm_GrayLevelVariance	x	x	x	
wavelet.HLL_gldm_LargeDependenceEmphasis	x	x		
wavelet.HLL_ngtdm_Complexity	x	x	x	x
wavelet.HLL_ngtdm_Contrast	x	x		
wavelet.HLH_firstorder_Entropy	x	x	x	x
wavelet.HLH_firstorder_10Percentile	x	x		x
wavelet.HLH_firstorder_90Percentile	x	x	x	x
wavelet.HLH_firstorder_InterquartileRange	x	x		x
wavelet.HLH_firstorder_MeanAbsoluteDeviation	x	x	x	x
wavelet.HLH_firstorder_RobustMeanAbsoluteDeviation	x	x	x	x
wavelet.HLH_firstorder_RootMeanSquared	x	x		x
wavelet.HLH_firstorder_Variance	x	x		x
wavelet.HLH_firstorder_Uniformity	x	x	x	
wavelet.HLH_glcm_ClusterProminence	x	x		x
wavelet.HLH_glcm_ClusterTendency	x	x	x	x
wavelet.HLH_glcm_Contrast	x	x	x	x
wavelet.HLH_glcm_DifferenceAverage	x	x	x	x
wavelet.HLH_glcm_DifferenceEntropy	x	x	x	x
wavelet.HLH_glcm_DifferenceVariance	x	x	x	x
wavelet.HLH_glcm_Imc2	x	x	x	
wavelet.HLH_glcm_InverseVariance	x	x		x
wavelet.HLH_glcm_SumEntropy	x	x	x	x
wavelet.HLH_glrlm_GrayLevelNonUniformityNormalized	x	x		
wavelet.HLH_glrlm_GrayLevelVariance	x	x		x
wavelet.HLH_glrlm_LongRunEmphasis	x	x	x	
wavelet.HLH_glrlm_RunEntropy	x	x	x	x
wavelet.HLH_glrlm_RunPercentage	x	x	x	
wavelet.HLH_glrlm_RunVariance	x	x	x	
wavelet.HLH_glrlm_ShortRunEmphasis	x	x	x	
wavelet.HLH_glszm_GrayLevelNonUniformityNormalized	x	x		x
wavelet.HLH_glszm_GrayLevelVariance	x	x		
wavelet.HLH_glszm_LargeAreaEmphasis	x	x	x	
wavelet.HLH_glszm_ZoneEntropy	x	x		x
wavelet.HLH_glszm_ZonePercentage	x	x		
wavelet.HLH_glszm_ZoneVariance	x	x	x	
wavelet.HLH_gldm_GrayLevelVariance	x	x		x
wavelet.HLH_gldm_LargeDependenceEmphasis	x	x		
wavelet.HLH_gldm_SmallDependenceEmphasis	x	x		
wavelet.HLH_ngtdm_Complexity	x	x	x	x
wavelet.HLH_ngtdm_Contrast	x	x		x
wavelet.HLH_ngtdm_Strength	x	x		

wavelet.HHL_firstorder_Entropy	x	x	x	x
wavelet.HHL_firstorder_10Percentile	x	x	x	x
wavelet.HHL_firstorder_90Percentile	x	x	x	x
wavelet.HHL_firstorder_InterquartileRange	x	x	x	x
wavelet.HHL_firstorder_MeanAbsoluteDeviation	x	x	x	x
wavelet.HHL_firstorder_RobustMeanAbsoluteDeviation	x	x	x	x
wavelet.HHL_firstorder_RootMeanSquared	x	x	x	x
wavelet.HHL_firstorder_Variance	x	x	x	x
wavelet.HHL_firstorder_Uniformity	x	x	x	x
wavelet.HHL_glcm_ClusterProminence	x	x	x	x
wavelet.HHL_glcm_ClusterTendency	x	x	x	x
wavelet.HHL_glcm_Contrast	x	x	x	x
wavelet.HHL_glcm_DifferenceAverage	x	x	x	x
wavelet.HHL_glcm_DifferenceEntropy	x	x	x	x
wavelet.HHL_glcm_DifferenceVariance	x	x	x	x
wavelet.HHL_glcm_InverseVariance	x	x	x	
wavelet.HHL_glcm_SumEntropy	x	x	x	x
wavelet.HHL_glrlm_GrayLevelNonUniformityNormalized	x	x	x	x
wavelet.HHL_glrlm_GrayLevelVariance	x	x	x	x
wavelet.HHL_glrlm_LongRunEmphasis	x	x	x	
wavelet.HHL_glrlm_RunEntropy	x	x	x	x
wavelet.HHL_glrlm_RunPercentage	x	x	x	
wavelet.HHL_glrlm_RunVariance	x	x	x	
wavelet.HHL_glrlm_ShortRunEmphasis	x	x	x	
wavelet.HHL_glszm_GrayLevelNonUniformityNormalized	x	x	x	x
wavelet.HHL_glszm_GrayLevelVariance	x	x	x	x
wavelet.HHL_glszm_LargeAreaEmphasis	x	x	x	
wavelet.HHL_glszm_ZonePercentage	x	x	x	
wavelet.HHL_glszm_ZoneVariance	x	x	x	
wavelet.HHL_gldm_DependenceEntropy	x	x	x	
wavelet.HHL_gldm_DependenceVariance	x	x	x	
wavelet.HHL_gldm_GrayLevelVariance	x	x	x	x
wavelet.HHL_gldm_LargeDependenceEmphasis	x	x	x	
wavelet.HHL_gldm_SmallDependenceEmphasis	x	x		
wavelet.HHL_gldm_SmallDependenceHighGrayLevelEmphasis	x	x		x
wavelet.HHH_firstorder_Entropy	x	x	x	x
wavelet.HHH_firstorder_10Percentile	x	x	x	x
wavelet.HHH_firstorder_90Percentile	x	x	x	x
wavelet.HHH_firstorder_InterquartileRange	x	x	x	x
wavelet.HHH_firstorder_MeanAbsoluteDeviation	x	x	x	x
wavelet.HHH_firstorder_RobustMeanAbsoluteDeviation	x	x	x	x

wavelet.HHH_firstorder_RootMeanSquared	x	x	x	x
wavelet.HHH_firstorder_Kurtosis	x	x		
wavelet.HHH_firstorder_Variance	x	x	x	x
wavelet.HHH_firstorder_Uniformity	x	x	x	
wavelet.HHH_glcm_ClusterProminence	x	x		
wavelet.HHH_glcm_ClusterTendency	x	x	x	x
wavelet.HHH_glcm_Contrast	x	x	x	x
wavelet.HHH_glcm_DifferenceAverage	x	x	x	x
wavelet.HHH_glcm_DifferenceEntropy	x	x	x	x
wavelet.HHH_glcm_DifferenceVariance	x	x	x	x
wavelet.HHH_glcm_InverseVariance	x	x		x
wavelet.HHH_glcm_SumEntropy	x	x	x	x
wavelet.HHH_glrlm_GrayLevelNonUniformityNormalized	x	x	x	x
wavelet.HHH_glrlm_GrayLevelVariance	x	x	x	x
wavelet.HHH_glrlm_LongRunEmphasis	x	x	x	
wavelet.HHH_glrlm_RunEntropy	x	x	x	x
wavelet.HHH_glrlm_RunVariance	x	x	x	
wavelet.HHH_glrlm_ShortRunEmphasis	x	x	x	
wavelet.HHH_glszm_GrayLevelNonUniformityNormalized	x	x		x
wavelet.HHH_glszm_GrayLevelVariance	x	x		x
wavelet.HHH_glszm_LargeAreaEmphasis	x	x	x	
wavelet.HHH_glszm_LargeAreaLowGrayLevelEmphasis	x	x		
wavelet.HHH_glszm_ZonePercentage	x	x	x	
wavelet.HHH_glszm_ZoneVariance	x	x	x	
wavelet.HHH_gldm_GrayLevelVariance	x	x		x
wavelet.HHH_ngtdm_Complexity	x	x		x
wavelet.HHH_ngtdm_Contrast	x	x	x	
wavelet.HHH_ngtdm_Strength	x	x		
wavelet.LLL_firstorder_10Percentile	x	x	x	x
wavelet.LLL_firstorder_90Percentile	x	x	x	x
wavelet.LLL_firstorder_Mean	x	x	x	
wavelet.LLL_firstorder_Median	x	x	x	
wavelet.LLL_firstorder_RootMeanSquared	x	x	x	x
wavelet.LLL_glrlm_GrayLevelNonUniformity	x	x	x	x
wavelet.LLL_glrlm_LongRunEmphasis	x	x	x	
wavelet.LLL_glrlm_RunPercentage	x	x	x	
wavelet.LLL_glrlm_RunVariance	x	x	x	
wavelet.LLL_glrlm_ShortRunEmphasis	x	x	x	
wavelet.LLL_glszm_GrayLevelNonUniformity	x	x	x	x
wavelet.LLL_glszm_LargeAreaEmphasis	x	x	x	
wavelet.LLL_glszm_SmallAreaEmphasis	x	x		
wavelet.LLL_glszm_ZonePercentage	x	x	x	x
wavelet.LLL_glszm_ZoneVariance	x	x	x	x
wavelet.LLL_gldm_GrayLevelNonUniformity	x	x	x	x

wavelet.LLL_gldm_LargeDependenceEmphasis	x	x	
wavelet.LLL_gldm_SmallDependenceEmphasis	x	x	
wavelet.LLL_ngtdm_Coarseness	x	x	x

Table S4. Comparison of radiomics features from preclinical and clinical inter-observer delineation variability studies. Reliable features with an ICC > 0.8 for preclinical lung contours (n=385) are compared with unreliable features with an ICC < 0.6 (n=54) determined from a clinical study of NSCLC contours. Overlapping features are highlighted in blue.

Preclinical Contours, ICC > 0.8	NSCLC, ICC < 0.6
**Original (unfiltered)**	
original_shape_LeastAxisLength	original_firstorder_10Percentile
original_firstorder_Minimum	original_firstorder_InterquartileRange
original_firstorder_10Percentile	original_firstorder_Kurtosis
original_firstorder_90Percentile	original_firstorder_Mean
original_firstorder_Mean	original_firstorder_MeanAbsoluteDeviation
original_firstorder_Median	original_firstorder_Median
original_firstorder_RootMeanSquared	original_firstorder_Minimum
original_glcm_DifferenceAverage	original_firstorder_RobustMeanAbsoluteDeviation
original_glcm_DifferenceEntropy	original_firstorder_RootMeanSquared
original_glcm_Idm	original_glcm_MCC
original_glcm_Id	original_gldm_DependenceEntropy
original_glcm_InverseVariance	original_glszm_GrayLevelVariance
original_glrlm_GrayLevelNonUniformity	original_glszm_ZoneEntropy
original_glrlm_LongRunEmphasis	original_ngtdm_Complexity
original_glrlm_RunLengthNonUniformityNormalized	original_shape_Compactness1
original_glrlm_RunPercentage	original_shape_Compactness2
original_glrlm_RunVariance	original_shape_Sphericity
original_glrlm_ShortRunEmphasis	
original_glszm_GrayLevelNonUniformity	
original_glszm_LargeAreaEmphasis	
original_glszm_SizeZoneNonUniformityNormalized	
original_glszm_SmallAreaEmphasis	
original_glszm_ZonePercentage	
original_glszm_ZoneVariance	
original_gldm_DependenceNonUniformityNormalized	
original_gldm_DependenceVariance	
original_gldm_GrayLevelNonUniformity	
original_gldm_SmallDependenceEmphasis	
**Wavelet (filtered)**	
wavelet.LLH_firstorder_Entropy	wavelet-HHH_firstorder_Mean
wavelet.LLH_firstorder_10Percentile	wavelet-HHH_firstorder_RootMeanSquared

wavelet.LLH_firstorder_90Percentile	wavelet-HHH_firstorder_Skewness
wavelet.LLH_firstorder_InterquartileRange	wavelet-HHH_glcm_ClusterShade
wavelet.LLH_firstorder_MeanAbsoluteDeviation	wavelet-HHH_gldm_LowGrayLevelEmphasis
wavelet.LLH_firstorder_RobustMeanAbsoluteDeviation	wavelet-HHH_glrlm_LongRunLowGrayLevelEmphasis
wavelet.LLH_firstorder_RootMeanSquared	wavelet-HHH_glrlm_LowGrayLevelRunEmphasis
wavelet.LLH_firstorder_Variance	wavelet-HHH_glrlm_ShortRunLowGrayLevelEmphasis
wavelet.LLH_firstorder_Uniformity	wavelet-HHH_glszm_LowGrayLevelZoneEmphasis
wavelet.LLH_glcm_ClusterTendency	wavelet-HHH_glszm_SmallAreaLowGrayLevelEmphasis
wavelet.LLH_glcm_Contrast	wavelet-HHL_firstorder_Mean
wavelet.LLH_glcm_DifferenceAverage	wavelet-HHL_glcm_ClusterShade
wavelet.LLH_glcm_DifferenceEntropy	wavelet-HLH_firstorder_Mean
wavelet.LLH_glcm_DifferenceVariance	wavelet-HLH_firstorder_RootMeanSquared
wavelet.LLH_glcm_JointEnergy	wavelet-HLH_firstorder_Skewness
wavelet.LLH_glcm_JointEntropy	wavelet-HLH_glcm_MCC
wavelet.LLH_glcm_Imc1	wavelet-LHH_firstorder_Mean
wavelet.LLH_glcm_Imc2	wavelet-LHH_firstorder_RootMeanSquared
wavelet.LLH_glcm_Idm	wavelet-LHH_firstorder_Skewness
wavelet.LLH_glcm_Id	wavelet-LHL_firstorder_Minimum
wavelet.LLH_glcm_InverseVariance	wavelet-LHL_glcm_Autocorrelation
wavelet.LLH_glcm_MaximumProbability	wavelet-LHL_glcm_JointAverage
wavelet.LLH_glcm_SumEntropy	wavelet-LHL_glcm_SumAverage
wavelet.LLH_glcm_SumSquares	wavelet-LHL_gldm_HighGrayLevelEmphasis
wavelet.LLH_glrlm_GrayLevelNonUniformityNormalized	wavelet-LHL_gldm_LargeDependenceLowGrayLevelEmphasis
wavelet.LLH_glrlm_GrayLevelVariance	wavelet-LHL_gldm_SmallDependenceHighGrayLevelEmphasis
wavelet.LLH_glrlm_LongRunEmphasis	wavelet-LHL_glrlm_HighGrayLevelRunEmphasis
wavelet.LLH_glrlm_RunEntropy	wavelet-LHL_glrlm_LongRunHighGrayLevelEmphasis
wavelet.LLH_glrlm_RunLengthNonUniformityNormalized	wavelet-LHL_glrlm_ShortRunHighGrayLevelEmphasis
wavelet.LLH_glrlm_RunPercentage	wavelet-LHL_glszm_HighGrayLevelZoneEmphasis
wavelet.LLH_glrlm_RunVariance	wavelet-LHL_glszm_SmallAreaHighGrayLevelEmphasis
wavelet.LLH_glrlm_ShortRunEmphasis	wavelet-LLH_glcm_Correlation
wavelet.LLH_glszm_GrayLevelNonUniformityNormalized	wavelet-LLH_gldm_LargeDependenceLowGrayLevelEmphasis

wavelet.LLH_glszm_GrayLevelVariance	wavelet-LLH_glszm_LargeAreaLow-GrayLevelEmphasis
wavelet.LLH_glszm_LargeAreaEmphasis	wavelet-LLL_glcm_MCC
wavelet.LLH_glszm_SizeZoneNonUniformi-tyNormalized	wavelet-LLL_gldm_LargeDepend-enceLowGrayLevelEmphasis
wavelet.LLH_glszm_SmallAreaEmphasis	wavelet-LLL_glszm_LargeAreaLow-GrayLevelEmphasis
wavelet.LLH_glszm_ZonePercentage	
wavelet.LLH_glszm_ZoneVariance	
wavelet.LLH_gldm_DependenceNonUni-formityNormalized	
wavelet.LLH_gldm_DependenceVariance	
wavelet.LLH_gldm_GrayLevelVariance	
wavelet.LLH_gldm_LargeDependenceEm-phasis	
wavelet.LLH_gldm_SmallDependenceEm-phasis	
wavelet.LHL_firstorder_Entropy	
wavelet.LHL_firstorder_10Percentile	
wavelet.LHL_firstorder_90Percentile	
wavelet.LHL_firstorder_Maximum	
wavelet.LHL_firstorder_Median	
wavelet.LHL_firstorder_InterquartileRange	
wavelet.LHL_firstorder_MeanAbsoluteDevi-ation	
wavelet.LHL_firstorder_RobustMeanAbso-luteDeviation	
wavelet.LHL_firstorder_RootMeanSquared	
wavelet.LHL_firstorder_Variance	
wavelet.LHL_firstorder_Uniformity	
wavelet.LHL_glcm_ClusterTendency	
wavelet.LHL_glcm_Contrast	
wavelet.LHL_glcm_Correlation	
wavelet.LHL_glcm_DifferenceAverage	
wavelet.LHL_glcm_DifferenceEntropy	
wavelet.LHL_glcm_DifferenceVariance	
wavelet.LHL_glcm_JointEnergy	
wavelet.LHL_glcm_JointEntropy	
wavelet.LHL_glcm_Imc1	
wavelet.LHL_glcm_Idm	
wavelet.LHL_glcm_Id	
wavelet.LHL_glcm_InverseVariance	
wavelet.LHL_glcm_MaximumProbability	
wavelet.LHL_glcm_SumEntropy	
wavelet.LHL_glcm_SumSquares	
wavelet.LHL_glrlm_GrayLevelNonUni-formity	

wavelet.LHL_glrlm_GrayLevelNonUniformityNormalized	
wavelet.LHL_glrlm_GrayLevelVariance	
wavelet.LHL_glrlm_LongRunEmphasis	
wavelet.LHL_glrlm_RunEntropy	
wavelet.LHL_glrlm_RunLengthNonUniformityNormalized	
wavelet.LHL_glrlm_RunPercentage	
wavelet.LHL_glrlm_RunVariance	
wavelet.LHL_glrlm_ShortRunEmphasis	
wavelet.LHL_glszm_GrayLevelNonUniformityNormalized	
wavelet.LHL_glszm_GrayLevelVariance	
wavelet.LHL_glszm_LargeAreaEmphasis	
wavelet.LHL_glszm_SizeZoneNonUniformityNormalized	
wavelet.LHL_glszm_SmallAreaEmphasis	
wavelet.LHL_glszm_ZonePercentage	
wavelet.LHL_glszm_ZoneVariance	
wavelet.LHL_gldm_DependenceEntropy	
wavelet.LHL_gldm_DependenceNonUniformityNormalized	
wavelet.LHL_gldm_DependenceVariance	
wavelet.LHL_gldm_GrayLevelNonUniformity	
wavelet.LHL_gldm_GrayLevelVariance	
wavelet.LHL_gldm_LargeDependenceEmphasis	
wavelet.LHL_gldm_SmallDependenceEmphasis	
wavelet.LHL_ngtdm_Contrast	
wavelet.LHH_firstorder_Entropy	
wavelet.LHH_firstorder_10Percentile	
wavelet.LHH_firstorder_90Percentile	
wavelet.LHH_firstorder_InterquartileRange	
wavelet.LHH_firstorder_MeanAbsoluteDeviation	
wavelet.LHH_firstorder_RobustMeanAbsoluteDeviation	
wavelet.LHH_firstorder_Variance	
wavelet.LHH_firstorder_Uniformity	
wavelet.LHH_glcm_ClusterTendency	
wavelet.LHH_glcm_Contrast	
wavelet.LHH_glcm_DifferenceAverage	
wavelet.LHH_glcm_DifferenceEntropy	
wavelet.LHH_glcm_DifferenceVariance	
wavelet.LHH_glcm_JointEnergy	

wavelet.LHH_glcm_JointEntropy	
wavelet.LHH_glcm_Idm	
wavelet.LHH_glcm_Id	
wavelet.LHH_glcm_InverseVariance	
wavelet.LHH_glcm_MaximumProbability	
wavelet.LHH_glcm_SumEntropy	
wavelet.LHH_glcm_SumSquares	
wavelet.LHH_glrlm_GrayLevelNonUniformityNormalized	
wavelet.LHH_glrlm_GrayLevelVariance	
wavelet.LHH_glrlm_LongRunEmphasis	
wavelet.LHH_glrlm_RunEntropy	
wavelet.LHH_glrlm_RunLengthNonUniformityNormalized	
wavelet.LHH_glrlm_RunPercentage	
wavelet.LHH_glrlm_RunVariance	
wavelet.LHH_glrlm_ShortRunEmphasis	
wavelet.LHH_glszm_GrayLevelNonUniformityNormalized	
wavelet.LHH_glszm_GrayLevelVariance	
wavelet.LHH_glszm_LargeAreaEmphasis	
wavelet.LHH_glszm_SizeZoneNonUniformityNormalized	
wavelet.LHH_glszm_SmallAreaEmphasis	
wavelet.LHH_glszm_ZonePercentage	
wavelet.LHH_glszm_ZoneVariance	
wavelet.LHH_gldm_DependenceEntropy	
wavelet.LHH_gldm_DependenceNonUniformityNormalized	
wavelet.LHH_gldm_DependenceVariance	
wavelet.LHH_gldm_GrayLevelVariance	
wavelet.LHH_gldm_LargeDependenceEmphasis	
wavelet.LHH_gldm_SmallDependenceEmphasis	
wavelet.HLL_firstorder_Entropy	
wavelet.HLL_firstorder_10Percentile	
wavelet.HLL_firstorder_90Percentile	
wavelet.HLL_firstorder_InterquartileRange	
wavelet.HLL_firstorder_MeanAbsoluteDeviation	
wavelet.HLL_firstorder_RobustMeanAbsoluteDeviation	
wavelet.HLL_firstorder_RootMeanSquared	
wavelet.HLL_firstorder_Variance	
wavelet.HLL_firstorder_Uniformity	
wavelet.HLL_glcm_ClusterProminence	

wavelet.HLL_glcm_ClusterTendency
wavelet.HLL_glcm_Contrast
wavelet.HLL_glcm_Correlation
wavelet.HLL_glcm_DifferenceAverage
wavelet.HLL_glcm_DifferenceEntropy
wavelet.HLL_glcm_DifferenceVariance
wavelet.HLL_glcm_JointEnergy
wavelet.HLL_glcm_JointEntropy
wavelet.HLL_glcm_Imc1
wavelet.HLL_glcm_Imc2
wavelet.HLL_glcm_Idm
wavelet.HLL_glcm_Id
wavelet.HLL_glcm_InverseVariance
wavelet.HLL_glcm_MaximumProbability
wavelet.HLL_glcm_SumEntropy
wavelet.HLL_glcm_SumSquares
wavelet.HLL_glrlm_GrayLevelNonUniformityNormalized
wavelet.HLL_glrlm_GrayLevelVariance
wavelet.HLL_glrlm_LongRunEmphasis
wavelet.HLL_glrlm_RunEntropy
wavelet.HLL_glrlm_RunLengthNonUniformityNormalized
wavelet.HLL_glrlm_RunPercentage
wavelet.HLL_glrlm_RunVariance
wavelet.HLL_glrlm_ShortRunEmphasis
wavelet.HLL_glszm_GrayLevelNonUniformityNormalized
wavelet.HLL_glszm_GrayLevelVariance
wavelet.HLL_glszm_LargeAreaEmphasis
wavelet.HLL_glszm_SizeZoneNonUniformityNormalized
wavelet.HLL_glszm_SmallAreaEmphasis
wavelet.HLL_glszm_ZoneEntropy
wavelet.HLL_glszm_ZonePercentage
wavelet.HLL_glszm_ZoneVariance
wavelet.HLL_gldm_DependenceEntropy
wavelet.HLL_gldm_DependenceNonUniformityNormalized
wavelet.HLL_gldm_DependenceVariance
wavelet.HLL_gldm_GrayLevelVariance
wavelet.HLL_gldm_LargeDependenceEmphasis
wavelet.HLL_gldm_SmallDependenceEmphasis
wavelet.HLL_ngtdm_Complexity
wavelet.HLL_ngtdm_Contrast

wavelet.HLH_firstorder_Entropy
wavelet.HLH_firstorder_10Percentile
wavelet.HLH_firstorder_90Percentile
wavelet.HLH_firstorder_Maximum
wavelet.HLH_firstorder_InterquartileRange
wavelet.HLH_firstorder_MeanAbsoluteDeviation
wavelet.HLH_firstorder_RobustMeanAbsoluteDeviation
wavelet.HLH_firstorder_RootMeanSquared
wavelet.HLH_firstorder_Skewness
wavelet.HLH_firstorder_Variance
wavelet.HLH_firstorder_Uniformity
wavelet.HLH_glcm_ClusterProminence
wavelet.HLH_glcm_ClusterTendency
wavelet.HLH_glcm_Contrast
wavelet.HLH_glcm_DifferenceAverage
wavelet.HLH_glcm_DifferenceEntropy
wavelet.HLH_glcm_DifferenceVariance
wavelet.HLH_glcm_JointEnergy
wavelet.HLH_glcm_JointEntropy
wavelet.HLH_glcm_Imc2
wavelet.HLH_glcm_Idm
wavelet.HLH_glcm_Id
wavelet.HLH_glcm_InverseVariance
wavelet.HLH_glcm_MaximumProbability
wavelet.HLH_glcm_SumEntropy
wavelet.HLH_glcm_SumSquares
wavelet.HLH_glrlm_GrayLevelNonUniformityNormalized
wavelet.HLH_glrlm_GrayLevelVariance
wavelet.HLH_glrlm_LongRunEmphasis
wavelet.HLH_glrlm_RunEntropy
wavelet.HLH_glrlm_RunLengthNonUniformityNormalized
wavelet.HLH_glrlm_RunPercentage
wavelet.HLH_glrlm_RunVariance
wavelet.HLH_glrlm_ShortRunEmphasis
wavelet.HLH_glszm_GrayLevelNonUniformityNormalized
wavelet.HLH_glszm_GrayLevelVariance
wavelet.HLH_glszm_LargeAreaEmphasis
wavelet.HLH_glszm_SizeZoneNonUniformityNormalized
wavelet.HLH_glszm_SmallAreaEmphasis
wavelet.HLH_glszm_ZoneEntropy
wavelet.HLH_glszm_ZonePercentage

wavelet.HLH_glszm_ZoneVariance
wavelet.HLH_gldm_DependenceEntropy
wavelet.HLH_gldm_DependenceNonUniformityNormalized
wavelet.HLH_gldm_DependenceVariance
wavelet.HLH_gldm_GrayLevelVariance
wavelet.HLH_gldm_LargeDependenceEmphasis
wavelet.HLH_gldm_SmallDependenceEmphasis
wavelet.HLH_ngtdm_Complexity
wavelet.HLH_ngtdm_Contrast
wavelet.HLH_ngtdm_Strength
wavelet.HHL_firstorder_Entropy
wavelet.HHL_firstorder_10Percentile
wavelet.HHL_firstorder_90Percentile
wavelet.HHL_firstorder_InterquartileRange
wavelet.HHL_firstorder_MeanAbsoluteDeviation
wavelet.HHL_firstorder_RobustMeanAbsoluteDeviation
wavelet.HHL_firstorder_RootMeanSquared
wavelet.HHL_firstorder_Variance
wavelet.HHL_firstorder_Uniformity
wavelet.HHL_glcm_ClusterProminence
wavelet.HHL_glcm_ClusterTendency
wavelet.HHL_glcm_Contrast
wavelet.HHL_glcm_DifferenceAverage
wavelet.HHL_glcm_DifferenceEntropy
wavelet.HHL_glcm_DifferenceVariance
wavelet.HHL_glcm_JointEnergy
wavelet.HHL_glcm_JointEntropy
wavelet.HHL_glcm_Imc2
wavelet.HHL_glcm_Idm
wavelet.HHL_glcm_Id
wavelet.HHL_glcm_InverseVariance
wavelet.HHL_glcm_MaximumProbability
wavelet.HHL_glcm_SumEntropy
wavelet.HHL_glcm_SumSquares
wavelet.HHL_glrlm_GrayLevelNonUniformityNormalized
wavelet.HHL_glrlm_GrayLevelVariance
wavelet.HHL_glrlm_LongRunEmphasis
wavelet.HHL_glrlm_RunEntropy
wavelet.HHL_glrlm_RunLengthNonUniformityNormalized
wavelet.HHL_glrlm_RunPercentage

wavelet.HHL_glrlm_RunVariance
wavelet.HHL_glrlm_ShortRunEmphasis
wavelet.HHL_glszm_GrayLevelNonUniformityNormalized
wavelet.HHL_glszm_GrayLevelVariance
wavelet.HHL_glszm_LargeAreaEmphasis
wavelet.HHL_glszm_SizeZoneNonUniformityNormalized
wavelet.HHL_glszm_SmallAreaEmphasis
wavelet.HHL_glszm_ZonePercentage
wavelet.HHL_glszm_ZoneVariance
wavelet.HHL_gldm_DependenceEntropy
wavelet.HHL_gldm_DependenceNonUniformityNormalized
wavelet.HHL_gldm_DependenceVariance
wavelet.HHL_gldm_GrayLevelVariance
wavelet.HHL_gldm_LargeDependenceEmphasis
wavelet.HHL_gldm_SmallDependenceEmphasis
wavelet.HHL_gldm_SmallDependenceHighGrayLevelEmphasis
wavelet.HHH_firstorder_Entropy
wavelet.HHH_firstorder_10Percentile
wavelet.HHH_firstorder_90Percentile
wavelet.HHH_firstorder_InterquartileRange
wavelet.HHH_firstorder_MeanAbsoluteDeviation
wavelet.HHH_firstorder_RobustMeanAbsoluteDeviation
wavelet.HHH_firstorder_RootMeanSquared
wavelet.HHH_firstorder_Kurtosis
wavelet.HHH_firstorder_Variance
wavelet.HHH_firstorder_Uniformity
wavelet.HHH_glcm_ClusterProminence
wavelet.HHH_glcm_ClusterTendency
wavelet.HHH_glcm_Contrast
wavelet.HHH_glcm_DifferenceAverage
wavelet.HHH_glcm_DifferenceEntropy
wavelet.HHH_glcm_DifferenceVariance
wavelet.HHH_glcm_JointEnergy
wavelet.HHH_glcm_JointEntropy
wavelet.HHH_glcm_Imc2
wavelet.HHH_glcm_Idm
wavelet.HHH_glcm_Id
wavelet.HHH_glcm_InverseVariance
wavelet.HHH_glcm_MaximumProbability

wavelet.HHH_glcm_SumEntropy
wavelet.HHH_glcm_SumSquares
wavelet.HHH_glrlm_GrayLevelNonUniformityNormalized
wavelet.HHH_glrlm_GrayLevelVariance
wavelet.HHH_glrlm_LongRunEmphasis
wavelet.HHH_glrlm_RunEntropy
wavelet.HHH_glrlm_RunLengthNonUniformityNormalized
wavelet.HHH_glrlm_RunPercentage
wavelet.HHH_glrlm_RunVariance
wavelet.HHH_glrlm_ShortRunEmphasis
wavelet.HHH_glszm_GrayLevelNonUniformityNormalized
wavelet.HHH_glszm_GrayLevelVariance
wavelet.HHH_glszm_LargeAreaEmphasis
wavelet.HHH_glszm_LargeAreaLowGrayLevelEmphasis
wavelet.HHH_glszm_SizeZoneNonUniformityNormalized
wavelet.HHH_glszm_SizeZoneNonUniformityNormalized
wavelet.HHH_glszm_SmallAreaEmphasis
wavelet.HHH_glszm_ZonePercentage
wavelet.HHH_glszm_ZoneVariance
wavelet.HHH_gldm_DependenceEntropy
wavelet.HHH_gldm_DependenceNonUniformityNormalized
wavelet.HHH_gldm_DependenceVariance
wavelet.HHH_gldm_GrayLevelVariance
wavelet.HHH_gldm_LargeDependenceEmphasis
wavelet.HHH_gldm_SmallDependenceEmphasis
wavelet.HHH_ngtdm_Complexity
wavelet.HHH_ngtdm_Contrast
wavelet.HHH_ngtdm_Strength
wavelet.LLL_firstorder_Minimum
wavelet.LLL_firstorder_10Percentile
wavelet.LLL_firstorder_90Percentile
wavelet.LLL_firstorder_Mean
wavelet.LLL_firstorder_Median
wavelet.LLL_firstorder_RootMeanSquared
wavelet.LLL_glrlm_GrayLevelNonUniformity
wavelet.LLL_glrlm_LongRunEmphasis

wavelet.LLL_glrlm_RunLengthNonUniformityNormalized
wavelet.LLL_glrlm_RunPercentage
wavelet.LLL_glrlm_RunVariance
wavelet.LLL_glrlm_ShortRunEmphasis
wavelet.LLL_glszm_GrayLevelNonUniformity
wavelet.LLL_glszm_LargeAreaEmphasis
wavelet.LLL_glszm_SmallAreaEmphasis
wavelet.LLL_glszm_ZonePercentage
wavelet.LLL_glszm_ZoneVariance
wavelet.LLL_gldm_DependenceNonUniformityNormalized
wavelet.LLL_gldm_DependenceVariance
wavelet.LLL_gldm_GrayLevelNonUniformity
wavelet.LLL_gldm_LargeDependenceEmphasis
wavelet.LLL_gldm_SmallDependenceEmphasis
wavelet.LLL_ngtdm_Coarseness

Table S5. Radiomics features stable across three independent studies which assessed different contouring methods of lung tissue and tumours and in both clinical and preclinical models. 271 features were identified including 14 unfiltered and 257 filtered features.

**Original (unfiltered)**	**Wavelet (filtered)**		
**original_shape_LeastAxisLength**	**wavelet.LLH_firstorder_Entropy**	wavelet.LHH_gldm_LargeDependenceEmphasis	wavelet.HHL_glszm_GrayLevelNonUniformityNormalized
original_firstorder_90Percentile	wavelet.LLH_firstorder_10Percentile	wavelet.HLL_firstorder_Entropy	wavelet.HHL_glszm_GrayLevelVariance
original_glcm_DifferenceAverage	wavelet.LLH_firstorder_90Percentile	wavelet.HLL_firstorder_10Percentile	wavelet.HHL_glszm_LargeAreaEmphasis
original_glcm_DifferenceEntropy	wavelet.LLH_firstorder_InterquartileRange	wavelet.HLL_firstorder_90Percentile	wavelet.HHL_glszm_ZonePercentage
original_glcm_InverseVariance	wavelet.LLH_firstorder_MeanAbsoluteDeviation	wavelet.HLL_firstorder_InterquartileRange	wavelet.HHL_glszm_ZoneVariance
original_glrlm_GrayLevelNonUniformity	wavelet.LLH_firstorder_RobustMeanAbsoluteDeviation	wavelet.HLL_firstorder_MeanAbsoluteDeviation	wavelet.HHL_gldm_DependenceEntropy

original_glrlm_LongRunEmphasis	wavelet.LLH_firstorder_RootMeanSquared	wavelet.HLL_firstorder_RobustMeanAbsoluteDeviation	wavelet.HHL_gldm_DependenceVariance
original_glrlm_RunPercentage	wavelet.LLH_firstorder_Variance	wavelet.HLL_firstorder_RootMeanSquared	wavelet.HHL_gldm_GrayLevelVariance
original_glrlm_ShortRunEmphasis	wavelet.LLH_firstorder_Uniformity	wavelet.HLL_firstorder_Variance	wavelet.HHL_gldm_LargeDependenceEmphasis
original_glszm_GrayLevelNonUniformity	wavelet.LLH_glcm_ClusterTendency	wavelet.HLL_firstorder_Uniformity	wavelet.HHL_gldm_SmallDependenceEmphasis
original_glszm_SizeZoneNonUniformityNormalized	wavelet.LLH_glcm_Contrast	wavelet.HLL_glcm_ClusterProminence	wavelet.HHL_gldm_SmallDependenceHighGrayLevelEmphasis
original_glszm_ZonePercentage	wavelet.LLH_glcm_DifferenceAverage	wavelet.HLL_glcm_ClusterTendency	wavelet.HHH_firstorder_Entropy
original_glszm_ZoneVariance	wavelet.LLH_glcm_DifferenceEntropy	wavelet.HLL_glcm_Contrast	wavelet.HHH_firstorder_10Percentile
original_gldm_GrayLevelNonUniformity	wavelet.LLH_glcm_DifferenceVariance	wavelet.HLL_glcm_DifferenceAverage	wavelet.HHH_firstorder_90Percentile
	wavelet.LLH_glcm_Imc1	wavelet.HLL_glcm_DifferenceEntropy	wavelet.HHH_firstorder_InterquartileRange
	wavelet.LLH_glcm_Imc2	wavelet.HLL_glcm_DifferenceVariance	wavelet.HHH_firstorder_MeanAbsoluteDeviation
	wavelet.LLH_glcm_InverseVariance	wavelet.HLL_glcm_Imc1	wavelet.HHH_firstorder_RobustMeanAbsoluteDeviation
	wavelet.LLH_glcm_SumEntropy	wavelet.HLL_glcm_Imc2	wavelet.HHH_firstorder_Kurtosis
	wavelet.LLH_glrlm_GrayLevelNonUniformityNormalized	wavelet.HLL_glcm_InverseVariance	wavelet.HHH_firstorder_Variance
	wavelet.LLH_glrlm_GrayLevelVariance	wavelet.HLL_glcm_SumEntropy	wavelet.HHH_firstorder_Uniformity
	wavelet.LLH_glrlm_LongRunEmphasis	wavelet.HLL_glrlm_Gray	wavelet.HHH_glcm_ClusterProminence

	LevelNonUniformityNormalized	
wavelet.LLH_glrlm_RunEntropy	wavelet.HLL_glrlm_GrayLevelVariance	wavelet.HHH_glcm_ClusterTendency
wavelet.LLH_glrlm_RunPercentage	wavelet.HLL_glrlm_LongRunEmphasis	wavelet.HHH_glcm_Contrast
wavelet.LLH_glrlm_RunVariance	wavelet.HLL_glrlm_RunEntropy	wavelet.HHH_glcm_DifferenceAverage
wavelet.LLH_glrlm_ShortRunEmphasis	wavelet.HLL_glrlm_RunPercentage	wavelet.HHH_glcm_DifferenceEntropy
wavelet.LLH_glszm_GrayLevelNonUniformityNormalized	wavelet.HLL_glrlm_RunVariance	wavelet.HHH_glcm_DifferenceVariance
wavelet.LLH_glszm_GrayLevelVariance	wavelet.HLL_glrlm_ShortRunEmphasis	wavelet.HHH_glcm_InverseVariance
wavelet.LLH_glszm_LargeAreaEmphasis	wavelet.HLL_glszm_GrayLevelNonUniformityNormalized	wavelet.HHH_glcm_SumEntropy
wavelet.LLH_glszm_SizeZoneNonUniformityNormalized	wavelet.HLL_glszm_GrayLevelVariance	wavelet.HHH_glrlm_GrayLevelNonUniformityNormalized
wavelet.LLH_glszm_SmallAreaEmphasis	wavelet.HLL_glszm_LargeAreaEmphasis	wavelet.HHH_glrlm_GrayLevelVariance
wavelet.LLH_glszm_ZonePercentage	wavelet.HLL_glszm_ZoneEntropy	wavelet.HHH_glrlm_LongRunEmphasis
wavelet.LLH_glszm_ZoneVariance	wavelet.HLL_glszm_ZonePercentage	wavelet.HHH_glrlm_RunEntropy
wavelet.LLH_gldm_GrayLevelVariance	wavelet.HLL_glszm_ZoneVariance	wavelet.HHH_glrlm_RunVariance
wavelet.LLH_gldm_SmallDependenceEmphasis	wavelet.HLL_gldm_GrayLevelVariance	wavelet.HHH_glrlm_ShortRunEmphasis
wavelet.LHL_firstorder_Entropy	wavelet.HLL_gldm_LargeDependenceEmphasis	wavelet.HHH_glszm_GrayLevelNonUniformityNormalized

	wavelet.LHL_firstorder_10Percentile	wavelet.HLL_ngtdm_Complexity	wavelet.HHH_glszm_GrayLevelVariance
	wavelet.LHL_firstorder_90Percentile	wavelet.HLL_ngtdm_Contrast	wavelet.HHH_glszm_LargeAreaEmphasis
	wavelet.LHL_firstorder_InterquartileRange	wavelet.HLH_firstorder_Entropy	wavelet.HHH_glszm_LargeAreaLowGrayLevelEmphasis
	wavelet.LHL_firstorder_MeanAbsoluteDeviation	wavelet.HLH_firstorder_10Percentile	wavelet.HHH_glszm_ZonePercentage
	wavelet.LHL_firstorder_RobustMeanAbsoluteDeviation	wavelet.HLH_firstorder_90Percentile	wavelet.HHH_glszm_ZoneVariance
	wavelet.LHL_firstorder_RootMeanSquared	wavelet.HLH_firstorder_InterquartileRange	wavelet.HHH_gldm_GrayLevelVariance
	wavelet.LHL_firstorder_Variance	wavelet.HLH_firstorder_MeanAbsoluteDeviation	wavelet.HHH_ngtdm_Complexity
	wavelet.LHL_firstorder_Uniformity	wavelet.HLH_firstorder_RobustMeanAbsoluteDeviation	wavelet.HHH_ngtdm_Contrast
	wavelet.LHL_glcm_ClusterTendency	wavelet.HLH_firstorder_Variance	wavelet.HHH_ngtdm_Strength
	wavelet.LHL_glcm_Contrast	wavelet.HLH_firstorder_Uniformity	wavelet.LLL_firstorder_10Percentile
	wavelet.LHL_glcm_Correlation	wavelet.HLH_glcm_ClusterProminence	wavelet.LLL_firstorder_90Percentile
	wavelet.LHL_glcm_DifferenceAverage	wavelet.HLH_glcm_ClusterTendency	wavelet.LLL_firstorder_Mean
	wavelet.LHL_glcm_DifferenceEntropy	wavelet.HLH_glcm_Contrast	wavelet.LLL_firstorder_Median
	wavelet.LHL_glcm_DifferenceVariance	wavelet.HLH_glcm_DifferenceAverage	wavelet.LLL_firstorder_RootMeanSquared
	wavelet.LHL_glcm_Imc1	wavelet.HLH_glcm_DifferenceEntropy	wavelet.LLL_glrlm_GrayLevelNonUniformity

wavelet.LHL_glcm_InverseVariance	wavelet.HLH_glcm_DifferenceVariance	wavelet.LLL_glrlm_LongRunEmphasis
wavelet.LHL_glcm_SumEntropy	wavelet.HLH_glcm_Imc2	wavelet.LLL_glrlm_RunPercentage
wavelet.LHL_glrlm_GrayLevelNonUniformity	wavelet.HLH_glcm_InverseVariance	wavelet.LLL_glrlm_RunVariance
wavelet.LHL_glrlm_GrayLevelNonUniformityNormalized	wavelet.HLH_glcm_SumEntropy	wavelet.LLL_glrlm_ShortRunEmphasis
wavelet.LHL_glrlm_GrayLevelVariance	wavelet.HLH_glrlm_GrayLevelNonUniformityNormalized	wavelet.LLL_glszm_GrayLevelNonUniformity
wavelet.LHL_glrlm_LongRunEmphasis	wavelet.HLH_glrlm_GrayLevelVariance	wavelet.LLL_glszm_LargeAreaEmphasis
wavelet.LHL_glrlm_RunEntropy	wavelet.HLH_glrlm_LongRunEmphasis	wavelet.LLL_glszm_SmallAreaEmphasis
wavelet.LHL_glrlm_RunPercentage	wavelet.HLH_glrlm_RunEntropy	wavelet.LLL_glszm_ZonePercentage
wavelet.LHL_glrlm_RunVariance	wavelet.HLH_glrlm_RunPercentage	wavelet.LLL_glszm_ZoneVariance
wavelet.LHL_glrlm_ShortRunEmphasis	wavelet.HLH_glrlm_RunVariance	wavelet.LLL_gldm_GrayLevelNonUniformity
wavelet.LHL_glszm_GrayLevelNonUniformityNormalized	wavelet.HLH_glrlm_ShortRunEmphasis	wavelet.LLL_gldm_LargeDependenceEmphasis
wavelet.LHL_glszm_GrayLevelVariance	wavelet.HLH_glszm_GrayLevelNonUniformityNormalized	wavelet.LLL_gldm_SmallDependenceEmphasis
wavelet.LHL_glszm_LargeAreaEmphasis	wavelet.HLH_glszm_GrayLevelVariance	wavelet.LLL_ngtdm_Coarseness
wavelet.LHL_glszm_ZonePercentage	wavelet.HLH_glszm_LargeAreaEmphasis	
wavelet.LHL_glszm_ZoneVariance	wavelet.HLH_glszm_ZoneEntropy	

wavelet.LHL_gldm_GrayLevelNonUniformity	wavelet.HLH_glszm_ZonePercentage
wavelet.LHL_gldm_GrayLevelVariance	wavelet.HLH_glszm_ZoneVariance
wavelet.LHL_gldm_LargeDependenceEmphasis	wavelet.HLH_gldm_GrayLevelVariance
wavelet.LHL_gldm_SmallDependenceEmphasis	wavelet.HLH_gldm_LargeDependenceEmphasis
wavelet.LHL_ngtdm_Contrast	wavelet.HLH_gldm_SmallDependenceEmphasis
wavelet.LHH_firstorder_Entropy	wavelet.HLH_ngtdm_Complexity
wavelet.LHH_firstorder_10Percentile	wavelet.HLH_ngtdm_Contrast
wavelet.LHH_firstorder_90Percentile	wavelet.HLH_ngtdm_Strength
wavelet.LHH_firstorder_InterquartileRange	wavelet.HHL_firstorder_Entropy
wavelet.LHH_firstorder_MeanAbsoluteDeviation	wavelet.HHL_firstorder_10Percentile
wavelet.LHH_firstorder_RobustMeanAbsoluteDeviation	wavelet.HHL_firstorder_90Percentile
wavelet.LHH_firstorder_Variance	wavelet.HHL_firstorder_InterquartileRange
wavelet.LHH_firstorder_Uniformity	wavelet.HHL_firstorder_MeanAbsoluteDeviation
wavelet.LHH_glcm_ClusterTendency	wavelet.HHL_firstorder_RobustMeanAbsoluteDeviation

wavelet.LHH_glcm_Contrast	wavelet.HHL_firstorder_RootMeanSquared
wavelet.LHH_glcm_DifferenceAverage	wavelet.HHL_firstorder_Variance
wavelet.LHH_glcm_DifferenceEntropy	wavelet.HHL_firstorder_Uniformity
wavelet.LHH_glcm_DifferenceVariance	wavelet.HHL_glcm_ClusterProminence
wavelet.LHH_glcm_InverseVariance	wavelet.HHL_glcm_ClusterTendency
wavelet.LHH_glcm_SumEntropy	wavelet.HHL_glcm_Contrast
wavelet.LHH_glrlm_GrayLevelNonUniformityNormalized	wavelet.HHL_glcm_DifferenceAverage
wavelet.LHH_glrlm_GrayLevelVariance	wavelet.HHL_glcm_DifferenceEntropy
wavelet.LHH_glrlm_LongRunEmphasis	wavelet.HHL_glcm_DifferenceVariance
wavelet.LHH_glrlm_RunEntropy	wavelet.HHL_glcm_InverseVariance
wavelet.LHH_glrlm_RunPercentage	wavelet.HHL_glcm_SumEntropy
wavelet.LHH_glrlm_RunVariance	wavelet.HHL_glrlm_GrayLevelNonUniformityNormalized
wavelet.LHH_glrlm_ShortRunEmphasis	wavelet.HHL_glrlm_GrayLevelVariance
wavelet.LHH_glszm_GrayLevelNonUniformityNormalized	wavelet.HHL_glrlm_LongRunEmphasis
wavelet.LHH_glszm_GrayLevelVariance	wavelet.HHL_glrlm_RunEntropy

wavelet.LHH_glszm_LargeAreaEmphasis	wavelet.HHL_glrlm_RunPercentage
wavelet.LHH_glszm_ZonePercentage	wavelet.HHL_glrlm_RunVariance
wavelet.LHH_gldm_GrayLevelVariance	wavelet.HHL_glrlm_ShortRunEmphasis
